# FABP4 secreted by M1-polarized macrophages promotes synovitis and angiogenesis to exacerbate rheumatoid arthritis

**DOI:** 10.1038/s41413-022-00211-2

**Published:** 2022-06-22

**Authors:** Dong Guo, Chuangxin Lin, Yuheng Lu, Hong Guan, Weizhong Qi, Hongbo Zhang, Yan Shao, Chun Zeng, Rongkai Zhang, Haiyan Zhang, Xiaochun Bai, Daozhang Cai

**Affiliations:** 1 Department of Joint Surgery, Center for Orthopedic Surgery, The Third Affiliated Hospital of Southern Medical University, Guangzhou, China; 2 Department of Orthopedics, Orthopedic Hospital of Guangdong Province, Academy of Orthopedics, Guangdong Province, The Third Affiliated Hospital of Southern Medical University, Guangzhou, China; 3 The Third School of Clinical Medicine, Southern Medical University, Guangzhou, China; 4 Guangdong Provincial Key Laboratory of Bone and Joint Degeneration Diseases, Guangzhou, China; 5 Department of Orthopedic Surgery, Shantou Central Hospital, Affiliated Shantou Hospital of Sun Yat-sen University, Shantou, China; 6 State Key Laboratory of Organ Failure Research, Department of Cell Biology, Southern Medical University School of Basic Medical Sciences, Guangzhou, China

**Keywords:** Bone, Pathogenesis

## Abstract

Increasing evidence shows that adipokines play a vital role in the development of rheumatoid arthritis (RA). Fatty acid-binding protein 4 (FABP4), a novel adipokine that regulates inflammation and angiogenesis, has been extensively studied in a variety of organs and diseases. However, the effect of FABP4 on RA remains unclear. Here, we found that FABP4 expression was upregulated in synovial M1-polarized macrophages in RA. The increase in FABP4 promoted synovitis, angiogenesis, and cartilage degradation to exacerbate RA progression in vivo and in vitro, whereas BMS309403 (a FABP4 inhibitor) and anagliptin (dipeptidyl peptidase 4 inhibitor) inhibited FABP4 expression in serum and synovial M1-polarized macrophages in mice to alleviate RA progression. Further studies showed that constitutive activation of mammalian target of rapamycin complex 1 (mTORC1) by TSC1 deletion specifically in the myeloid lineage regulated FABP4 expression in macrophages to exacerbate RA progression in mice. In contrast, inhibition of mTORC1 by ras homolog enriched in brain (Rheb1) disruption specifically in the myeloid lineage reduced FABP4 expression in macrophages to attenuate RA development in mice. Our findings established an essential role of FABP4 that is secreted by M1-polarized macrophages in synovitis, angiogenesis, and cartilage degradation in RA. BMS309403 and anagliptin inhibited FABP4 expression in synovial M1-polarized macrophages to alleviate RA development. Hence, FABP4 may represent a potential target for RA therapy.

## Introduction

Rheumatoid arthritis (RA) is the most common form of chronic inflammatory arthritis and is primarily characterized by joint destruction. RA is a complex pathological process involving persistent synovitis, angiogenesis, cartilage degradation, and bone erosion that affects approximately 1% of the global population.^1^ The pathogenesis of RA is highly complex and remains incompletely understood.^2^ Without early and sufficient intervention, persistent RA development leads to irreversible disability and influences patient quality of life. However, there is currently no effective clinical means of curing RA. Therefore, it is necessary to further study RA pathogenesis and drug targets.

Synovitis and angiogenesis are key pathological changes during the pathogenesis and persistent progression of RA that lead to synovial tissue hyperplasia and the invasion of multiple types of synovial cells.^3^ The synovium is the primary site of inflammation associated with RA, and immune cells such as macrophages secrete IL-1β and TNFα to exacerbate synovitis and promote RA progression.^4^ Angiogenesis gradually occurs with persistent synovitis, which causes more inflammatory macrophages to be recruited to the synovium and secrete more inflammatory factors to exacerbate synovitis.^5^ Therefore, to better alleviate the progression of RA, we hypothesize that both synovitis and angiogenesis need to be inhibited. Moreover, the specific molecular mechanisms of synovitis and angiogenesis in RA are still unclear.

Recent evidence has demonstrated a relationship between adipokines, inflammation, and angiogenesis.^6^ Adipokines promote inflammation and angiogenesis in type 2 diabetes mellitus and breast cancer.^7,8^ The importance of adipokines in the pathophysiology of RA has been reported; however, the role of adipokines in the progression of RA remains unclear.^9^ Andrés Cerezo et al. identified the novel adipokine fatty acid-binding protein 4 (FABP4), which was upregulated in patients with RA.^10^ Furthermore, Chen et al. reported that the increase in FABP4 expression was proportional to the severity of RA.^11^ FABP4 is a small cytosolic lipid-binding protein with a molecular weight of approximately 15 kDa and is a member of the cytoplasmic fatty acid binding protein (FABP) multigene family. FABP4 is secreted by macrophages and adipocytes and acts on multiple integrated pathways to regulate inflammation, promote proliferation and angiogenesis, and contribute to the pathogenesis of cancer and immunometabolic diseases (e.g., diabetes mellitus and atherosclerosis). In addition, BMS309403 (a FABP4 inhibitor) reduces inflammation and angiogenesis in diabetes and atherosclerosis.^12^ Previous studies have reported that M1-polarized macrophages in the synovium promote inflammation and angiogenesis in RA;^13^ however, the secretion of FABP4 by synovial macrophages and its role in RA progression remain unclear. In this study, we found that FABP4 secretion by synovial M1-polarized macrophages was upregulated. The increase in FABP4 was regulated by the mammalian target of rapamycin complex 1 (mTORC1) pathway to promote synovitis, angiogenesis, and cartilage degeneration, which exacerbated the severity of experimental RA. In contrast, BMS309403 prevented experimental RA progression by inhibiting FABP4 expression in the synovial M1-polarized macrophages of mice. Thus, FABP4 may represent a novel therapeutic target for the treatment of RA.

## Results

### FABP4 is upregulated in synovial M1-polarized macrophages in RA

**Fig. 1:**
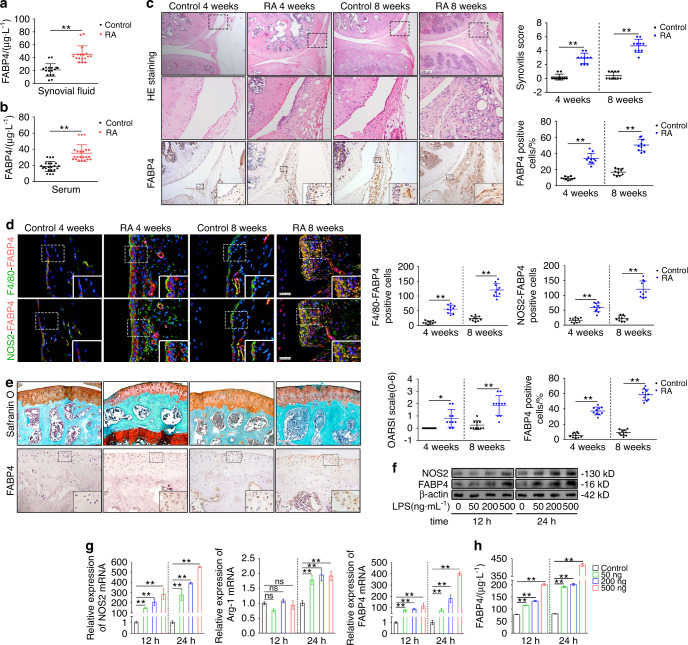
The expression of FABP4 in the RA mouse model, BMDMs and RA patients. **a**, **b** FABP4 concentrations in the synovial fluid (**a**) and serum (**b**) of controls and RA patients (*n* = 8 per group) were assessed by ELISA. **C** Representative images and quantification of HE staining and FABP4 immunohistochemical staining in the synovium of control and C57BL/6 J mice at 4 and 8 weeks after AIA modeling (*n* = 10 per group). Scale bars: 50 μm, 100 μm and 200 μm. **d** Representative images and quantitative analysis of coimmunostaining of FABP4 with F4/80 or NOS2 in the synovium of control and C57BL/6 J mice at 4 and 8 weeks after AIA modeling (*n* = 10 per group). Scale bars: 12.5 μm. **e** Representative images and quantification of safranin O and fast green staining and FABP4 immunohistochemical staining in knee cartilage from controls and C57BL/6 J mice at 4 and 8 weeks after AIA modeling (*n* = 10 per group). Scale bars: 50 μm and 100 μm. **f** Western blot showing FABP4 and NOS2 in BMDMs stimulated with 50, 200, or 500 ng·mL^−1^ LPS for 12 h or 24 h. **g** Quantitative PCR analysis of NOS2, Arg-1, and FABP4 mRNA expression in BMDMs (*n* = 3 per group). **h** FABP4 concentrations in the supernatant of BMDMs stimulated with 50, 200, or 500 ng·mL^−1^ LPS for 12 h or 24 h (*n* = 3 per group) were assessed by ELISA. Student’s *t*-test or one-way analysis of variance (ANOVA) and Tukey’s multiple comparison test. ^*^P < 0.05, ^**^P < 0.01; ns, no significance. The data are shown as the mean ± SEM

In this study, we first examined the pathological characteristics of the RA synovium by histological staining to score synovitis in human RA synovial tissue. High levels of synovial hyperplasia and abundant cell infiltration were observed in human RA synovial tissue, which were combined with significantly higher synovitis scores than those of the controls (Fig. S1A, B). We further measured FABP4 expression in the synovium, synovial fluid, serum and cartilage of RA patients. Compared to that in the controls, immunohistochemical (IHC) analysis revealed that FABP4 staining in the RA synovium was significantly increased (Fig. S1A, C). Western blot analysis showed that the protein expression of FABP4 in RA synovial tissue was markedly higher than that in control synovial tissue (Fig. S1D). The concentration of FABP4 in RA synovial fluid and serum was significantly elevated compared to that in control synovial fluid and serum (Fig. 1a, b). To determine whether FABP4 was expressed in M1-polarized macrophages, the colocalization of FABP4 with F4/80 (a macrophage marker) or NOS2 (an M1-like macrophage marker) was examined in human RA synovial tissue (Fig. S1E). An increase in M1-polarized macrophages and elevated colocalization of FABP4 with F4/80 or NOS2 were observed, which suggested the upregulation of FABP4 in M1-polarized macrophages of human RA synovial tissue. Then, we examined the expression of FABP4 in cartilage from the tibial plateaus of RA patients and controls. Immunohistochemical staining revealed that FABP4 was increased in the damaged cartilage of the tibial plateaus of RA patients who underwent total knee arthroplasty compared to control cartilage samples from the lateral tibial plateaus of OA patients, which were only slightly damaged (Fig. S1F, G). Interestingly, the concentration of FABP4 in the supernatant of chondrocytes from RA patients was similar to that in control samples (Fig. S1H). The expression of FABP4 in adipose tissue was not significantly different between RA patients and controls (Fig. S1I).

We then examined histological characteristics and FABP4 expression in the knee joints of male C57BL/6 J mice at 4, 8, and 12 weeks after antigen-induced arthritis (AIA) establishment. Hematoxylin and eosin (HE) staining showed that the RA group exhibited obvious synovial hyperplasia, increased cell infiltration, and higher synovitis scores than the control (Fig. 1c and Fig. S2A), which was consistent with an increase in FABP4 expression in mice with AIA (Fig. 1c and Fig. S2B). The level of serum FABP4 in RA mice was higher than that in control mice (Fig. S2C). These data demonstrated that FABP4 expression was upregulated in the synovium and serum of the RA mouse model. Next, we determined whether FABP4 was expressed in synovial M1-polarized macrophages in the RA mouse model by examining the colocalization of FABP4 with F4/80 or NOS2 in the synovium (Fig. 1d and Fig. S2B). Increased colocalization of FABP4 with F4/80 or NOS2 indicated elevated FABP4 expression in synovial M1-polarized macrophages in the RA mouse model. We then analyzed the expression of FABP4 in cartilage during RA progression in the RA mouse model. Similarly, few FABP4-expressing chondrocytes were observed in normal cartilage, while FABP4 was progressively upregulated and associated with increased cartilage damage in RA mice (Fig. 1e and Fig. S2D).

To measure the expression of FABP4 in M1-polarized macrophages in vitro, we induced bone marrow-derived macrophages (BMDMs) to undergo M1 polarization with lipopolysaccharide (LPS). We found that the protein and mRNA expression levels of FABP4 and NOS2 in LPS-induced BMDMs were increased in a concentration- and time-dependent manner. The mRNA expression of Arg-1 in BMDMs was similar in the control and LPS-treated groups at 12 h after LPS stimulation and increased at 24 h after LPS administration (Fig. 1f, g). The concentration of FABP4 in M1-polarized macrophage supernatant was also elevated in a concentration- and time-dependent manner (Fig. 1h). Taken together, these results suggested that M1-polarized macrophages secreted FABP4 in the context of RA.

### FABP4 regulates tube formation, proliferation, migration, and invasion

**Fig. 2:**
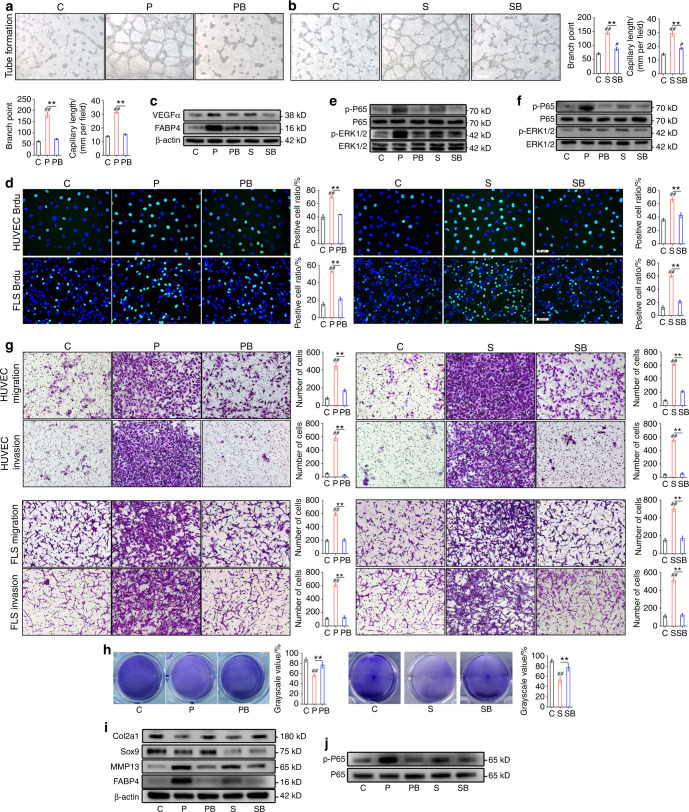
The effect of FABP4 and M1-polarized macrophage supernatant on HUVECs, FLSs, and chondrocytes in vitro. **a**, **b** Tube formation assay and quantification of HUVECs cultured with vehicle (C), rhFABP4 (P), rhFABP4 + BMS309403 (PB), M1-polarized macrophage supernatant (S), or M1-polarized macrophage supernatant+BMS309403 (SB) (*n* = 3 per group). Scale bar: 100 µm. **c** Immunoblot analysis of FABP4 and VEGFα in HUVECs cultured with C, P, PB, S, or SB for 24 h. **d**, **g** Representative images and quantification of BrdU (green) immunofluorescence (**d**) and Transwell assays (**g**) in HUVECs and FLSs treated with C, P, PB, S, or SB for 24 h (*n* = 3 per group). Scale bar: 25 µm, 100 µm. **e**, **f** Immunoblot analysis of ERK1/2, p-ERK1/2, P65, and p-P65 in HUVECs (**e**) and FLSs (**f**) treated with C, P, PB, S, or SB for 1 h. **h** Toluidine blue staining of ATDC5 cells treated with C, P, PB, S, or SB for 24 h (*n* = 3 per group). **i** Immunoblot analysis of FABP4, MMP13, Sox9, and Col2a1 in primary chondrocytes treated with C, P, PB, S, or SB for 24 h. **j** Immunoblot analysis of P65 and p-P65 in primary chondrocytes treated with C, P, PB, S, or SB for 1 h. One-way ANOVA and Tukey’s multiple comparison test. ^*^P < 0.05, ^**^P < 0.01; ^#^*P* < 0.05, ^##^*P* < 0.01 compared to the control. The data are shown as the mean ± SEM

Angiogenesis in synovial tissue is a key pathological event in RA progression. To determine the role of FABP4 in human umbilical vein endothelial cell (HUVEC) tube formation, HUVECs were treated with recombinant human FABP4 (rhFABP4) (P), rhFABP4 plus BMS309403 (a FABP4 inhibitor) (PB), or the control vehicle (C). To determine whether FABP4 in the supernatant of M1-polarized macrophages plays a key role, we treated HUVECs with supernatant (S), supernatant plus BMS309403 (SB), or the control vehicle (C). We measured the toxicity of BMS309403 to HUVECs and fibroblast-like synoviocytes (FLSs) by CCK-8 assays. We found that at the concentration used (20 μmol·L^−1^), BMS309403 had no toxicity toward HUVECs or FLSs at 24 h, 48 h and 72 h (Fig. S3A, B). Tube formation indued by rhFABP4 or M1-polarized macrophage supernatant was significantly enhanced compared to that induced by the control, and this effect was inhibited by BMS309403 (Fig. 2a, b). Furthermore, rhFABP4 or M1-polarized macrophage supernatant promoted vascular endothelial growth factor α (VEGFα) protein expression in HUVECs, whereas BMS309403 significantly inhibited this effect (Fig. 2c).^14^ To further demonstrate the role of FABP4 in tube formation, lentivirus-mediated shRNA targeting FABP4 was transfected into HUVECs (Fig. S3C). We found that the enhanced tube formation stimulated by rhFABP4 was mitigated by FABP4 knockdown (Fig. S3D). These data demonstrate that FABP4 is a main effector cytokine in the M1-polarized macrophage supernatant that regulates HUVEC tube formation by activating VEGFα expression.

Synovial hyperplasia and infiltration are important pathological changes in RA and include the proliferation, migration, and invasion of HUVECs and FLSs. Next, we sought to elucidate the role of FABP4 in the proliferation, migration, and invasion of HUVECs and FLSs. HUVECs and FLSs were treated with rhFABP4, rhFABP4 plus BMS309403, M1-polarized macrophage supernatant, M1-polarized macrophage supernatant plus BMS309403, or the control vehicle. A substantial increase was observed in the number of BrdU^+^ cells among HUVECs and FLSs treated with rhFABP4 or M1-polarized macrophage supernatant compared to those that were treated with the control, and this effect was markedly reversed by BMS309403 (Fig. 2d). These findings indicated that rhFABP4 or M1-polarized macrophage supernatant promoted HUVEC and FLS proliferation. Additionally, the CCK-8 assay results further confirmed the effect of rhFABP4 or M1-polarized macrophage supernatant on the proliferation of HUVECs and FLSs (Fig. S3E, F). It has previously been reported that the MAPK pathway regulates the proliferation of HUVECs and FLSs.^15,16^ We found that rhFABP4 or M1-polarized macrophage supernatant promoted ERK1/2 (a downstream effector of MAPK) phosphorylation in HUVECs (Fig. 2e) and FLSs (Fig. 2f), while BMS309403 significantly inhibited this effect. These data suggest that FABP4 is the main effector cytokine by which M1-polarized macrophages regulate the proliferation of HUVECs and FLSs partially through the MAPK pathway. Compared to cells treated with the control, HUVECs and FLSs treated with rhFABP4 or M1-polarized macrophage supernatant exhibited obvious migration and invasion, as indicated by the increased numbers of cells (Fig. 2g) and the elevated percentage of wound healing (Fig. S3G, H). BMS309403 significantly inhibited the migration and invasion of HUVECs and FLSs induced by rhFABP4 or M1-polarized macrophage supernatant (Fig. 2g and Fig. S3G, H). These data demonstrate that FABP4, the key effector cytokine in M1-polarized macrophage supernatant, promotes HUVEC and FLS migration and invasion.

### FABP4 promotes inflammatory cytokine production by HUVECs and FLSs

Since the presence of inflammatory factors is known to exacerbate the progression of RA,^17,18^ we next identified the role of FABP4 in the release of inflammatory cytokines by HUVECs and FLSs. The production of inflammatory cytokines, such as IL-1β, IL-6 and IL-18, was increased in HUVECs and FLSs following treatment with rhFABP4 or M1-polarized macrophage supernatant compared to the control, and this effect was inhibited by BMS309403 (Fig. S4A, B). It is well known that the NF-κB pathway regulates the release of inflammatory cytokines.^19,20^ We found that rhFABP4 or M1-polarized macrophage supernatant substantially enhanced P65 (a downstream effector of NF-κB) phosphorylation in HUVECs (Fig. 2e) and FLSs (Fig. 2f), whereas BMS309403 significantly inhibited this effect. These data indicate that FABP4 is the main effector cytokine in the M1-polarized macrophage supernatant and promotes the production of inflammatory cytokines by HUVECs and FLSs partially through the NF-κB pathway.

### FABP4 disrupts chondrocyte homeostasis

Cartilage destruction is a main pathological feature of RA progression. To explore the role of FABP4 in chondrocyte homeostasis during RA progression, we treated chondrocytes with rhFABP4, rhFABP4 plus BMS309403, M1-polarized macrophage supernatant, M1-polarized macrophage supernatant plus BMS309403, or the control vehicle. Toluidine blue staining of chondrocytes treated with rhFABP4 or M1-polarized macrophage supernatant was lighter than that in the control group, and BMS309403 significantly reversed this effect (Fig. 2h). This finding revealed that rhFABP4 or M1-polarized macrophage supernatant promoted the degradation of acid mucopolysaccharide. Furthermore, Western blot analysis showed that Col2a1 (type 2 collagen) and Sox9 protein production was downregulated, whereas matrix metalloproteinase 13 (MMP13) protein production was upregulated in chondrocytes treated with rhFABP4 or M1-polarized macrophage supernatant (Fig. 2i). We found that rhFABP4 or M1-polarized macrophage supernatant substantially elevated P65 phosphorylation in chondrocytes, while the increase in P65 phosphorylation induced by rhFABP4 or M1-polarized macrophage supernatant was markedly reversed by BMS309403 treatment (Fig. 2j). These data demonstrated that the effects of FABP4 on chondrocytes were partly mediated by activation of the NF-κB pathway. The RT–qPCR results showed that Col2a1 and aggrecan (ACAN) mRNA production was reduced, whereas MMP13 and disintegrin and metalloproteinase with thrombospondin motifs 5 (ADAMTS5) mRNA production was increased in chondrocytes treated with rhFABP4 or M1-polarized macrophage supernatant (Fig. S4C). On the other hand, BMS309403 treatment significantly reversed chondrocyte metabolic disruption induced by rhFABP4 or M1-polarized macrophage supernatant (Fig. 2h, i and Fig. S4C). Taken together, these results show that FABP4, which is a key effector in M1-polarized macrophage supernatant, disrupts chondrocyte homeostasis by enhancing chondrocyte catabolism and suppressing chondrocyte anabolism.

### FABP4 promotes synovitis, angiogenesis, and cartilage degradation to exacerbate RA progression

**Fig. 3:**
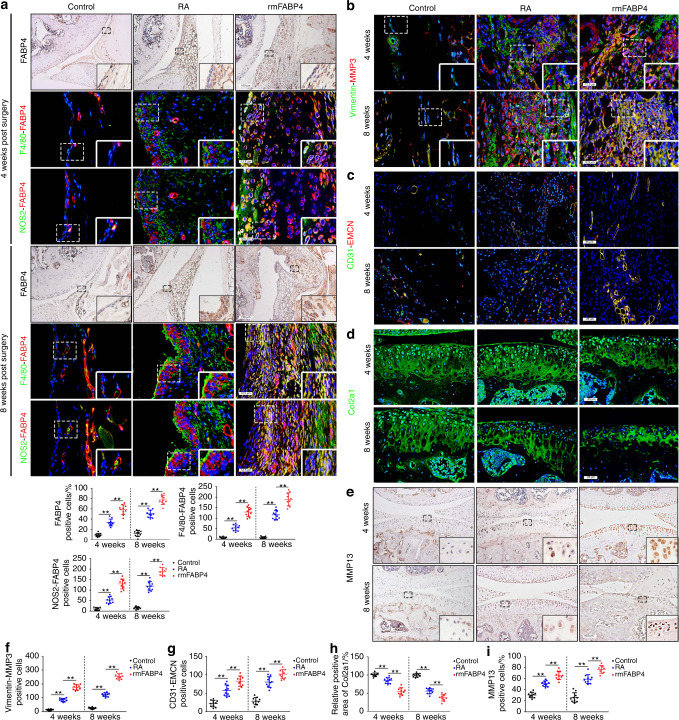
Recombinant FABP4 exacerbates the development of RA in C57BL/6 J mice. **a** Representative images and quantitative analysis of FABP4 were assessed by immunohistochemical staining and coimmunostaining of FABP4 with F4/80 or NOS2 in the synovium of control and RA mice treated with vehicle or rmFABP4 for 4 and 8 weeks (*n* = 10 per group). Scale bars: 12.5 µm and 100 µm. **b**–**i** Representative images and quantification of Vimentin and MMP3 coimmunostaining (**b**, **f**), CD31 and EMCN coimmunostaining (**c**, **g**), Col2a1 immunofluorescence staining (**d**, **h**), and MMP13 immunohistochemical staining (**e**, **i**) in the knee joints of control and RA mice treated with vehicle or rmFABP4 for 4 and 8 weeks (*n* = 10 per group). Scale bars: 12.5 µm, 25 µm, and 100 µm. One-way ANOVA and Tukey’s multiple comparison test. ^**^P < 0.01. The data are shown as the mean ± SEM

Next, we explored the role of FABP4 in vivo using intra-articular injection of recombinant murine FABP4 (rmFABP4) for 4 and 8 weeks. Increased FABP4 expression in the synovial membrane was observed, and FABP4 colocalization with F4/80 or NOS2 was also elevated in RA mice treated with rmFABP4 compared to vehicle-treated mice (Fig. 3a). These data suggested the upregulation of FABP4 expression in M1-polarized macrophages caused by intra-articular injection of rmFABP4. To explore the role of FABP4 in macrophage proliferation, BMDMs were treated with rmFABP4 with/without BMS309403. Compared to that in the control group, an increased number of BrdU^+^ cells among BMDMs treated with rmFABP4 was observed, and this could be inhibited by BMS309403 (Fig. S5A). In addition, the proportion of M1 macrophages (CD86^+^CD206^−^) and the ratios of M1/M2 cells in the LPS group, FABP4 group and LPS + FABP4 group were significantly higher than those in the control group, while the proportion of M2 macrophages (CD86^−^CD206^+^) was significantly reduced. Moreover, BMS309403 effectively reversed the increase in the proportion of M1 macrophages, the elevation in the M1/M2 ratio, and the decrease in the proportion of M2 macrophages caused by rmFABP4. The proportion of M1 macrophages and the ratio of M1/M2 cells in the IL4 group were significantly less than those in the control group, while the proportion of M2 macrophages was increased. Interestingly, the increase in M2 macrophage polarization, decrease in M1 macrophage proportion, and decrease in the M1/M2 ratio induced by IL4 were reversed by rhFABP4 treatment (Fig. S5B). Then, the role of rmFABP4 was assessed by examining the histopathological characteristics of the RA mouse model. Increased levels of synovial hyperplasia, cellular infiltration and elevated synovitis scores were observed in mice treated with rmFABP4 compared to those in the vehicle group (Fig. S6A). Mice treated with rmFABP4 exhibited increased MMP3 expression and colocalization with Vimentin (a synovial fibroblast marker) compared to mice treated with the vehicle (Fig. 3b, f and Fig. S6B). This finding indicated that there was an increase in synovial fibroblasts and enhanced invasion. Following rmFABP4 treatment, H-type vessels, which are characterized by the colocalization of CD31 and EMCN, were significantly increased (Fig. 3c, g), indicating an increase in angiogenesis.^21^ Mice treated with rmFABP4 exhibited higher OARSI scores (Fig. S6C), decreases in Col2a1-positive areas (Fig. 3d, h and Fig. S6D) and increases in MMP13 expression (Fig. 3e, i) in cartilage than mice treated with the vehicle, which indicated more severe cartilage degradation. An obvious increase in osteoclasts was observed in mice that were treated with rmFABP4 for 8 weeks compared to mice in the vehicle group, and there was no significant difference between the rmFABP4 group and vehicle groups at 4 weeks (Fig. S6E). To determine the expression and effect of FABP4 in the advanced-stage RA mouse model, we examined the histopathological characteristics of mice at 12 weeks after AIA. A similar effect was observed in mice at 12 weeks after AIA (Fig. S2D–K). These data suggested that rmFABP4 promoted synovitis, the angiogenesis of H-type vessels, and cartilage degradation in an RA mouse model.

### FABP4 upregulation in M1-polarized macrophages is regulated by the mTORC1 pathway and exacerbates RA development

**Fig. 4:**
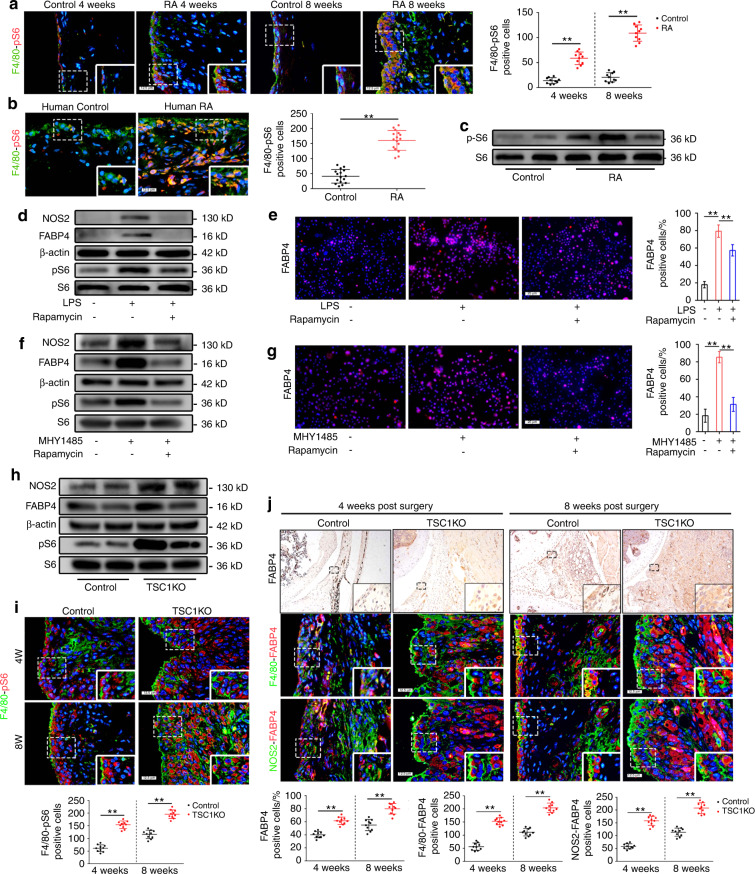
Activation of the mTORC1 pathway enhances the secretion of FABP4 by M1-polarized macrophages to exacerbate RA progression. **a**, **b** Representative images and quantification of F4/80 and pS6 coimmunostaining in the mouse (*n* = 10 per group) (**a**) and human synovium (*n* = 16 per group) (**b**) of the control and RA groups. Scale bar: 12.5 µm. **c** Western blot showing S6 and pS6 in human synovial tissue. **d** Western blot showing FABP4, NOS2, S6 and pS6 in BMDMs treated with LPS or rapamycin. **e** Representative immunofluorescence staining images and quantitative analysis of FABP4 in BMDMs stimulated with LPS or rapamycin (*n* = 3 per group). Scale bar: 100 µm. **f** Western blot showing FABP4, NOS2, S6 and pS6 in BMDMs treated with MHY1485 or rapamycin. **g** Representative immunofluorescence staining images and quantitative analysis of FABP4 in BMDMs treated with MHY1485 or rapamycin (*n* = 3 per group). Scale bar: 100 µm. **h** Western blot showing FABP4, NOS2, S6 and pS6 in BMDMs from control and TSC1KO mice. **i** Representative images and quantification of F4/80 and pS6 coimmunostaining in the synovium of wild-type and TSC1KO mice at 4 and 8 weeks after AIA modeling (*n* = 10 per group). Scale bar: 12.5 µm. **j** Representative images and quantification of FABP4 immunohistochemical staining and coimmunostaining of FABP4 with F4/80 or NOS2 in the synovium of control and TSC1KO mice at 4 and 8 weeks after AIA modeling (*n* = 10 per group). Scale bars: 12.5 µm and 100 µm. **f–j** Representative images (**f**) and quantification of Vimentin and MMP3 coimmunostaining (**g**), CD31 and EMCN coimmunostaining (**h**), Col2a1 immunofluorescence staining (**i**), and MMP13 immunohistochemical staining (**j**) in the knee joints of controls and TSC1KO mice at 4 and 8 weeks after AIA modeling (*n* = 10 per group). Scale bar: 12.5 µm, 25 µm, and 100 µm. Student’s *t*-test or one-way ANOVA and Tukey’s multiple comparison test. ^**^P < 0.01. The data are shown as the mean ± SEM

Previous studies have shown that the mTOR pathway is activated at sites of inflammation in RA and that activation of the mTORC1 pathway regulates FABP4 expression in hemangiomas;^22,23^ however, it remains unclear whether activation of the mTORC1 pathway regulates FABP4 expression in RA. Enhanced phosphorylation of S6 (S235/236) (a downstream effector of mTORC1) and its colocalization with F4/80 was observed in the synovium of mice that underwent AIA, whereas phosphorylated S6-positive macrophages were virtually undetectable in the control synovium (Fig. 4a and Fig. S2L). Activation of the mTORC1 pathway in macrophages was also examined in human RA synovial tissue (Fig. 4b, c). To investigate the activation of the mTOR pathway and the expression of FABP4 in macrophages in vitro, BMDMs were stimulated with LPS with or without rapamycin (a specific inhibitor of the mTOR pathway). BMDMs that were stimulated with LPS exhibited obvious upregulation of p-S6, NOS2, and FABP4, and this effect was significantly mitigated by rapamycin (Fig. 4d, e). Next, to explore whether the mTOR pathway regulates FABP4 expression in macrophages, BMDMs were treated with MHY1485 (a potent mTOR activator) with or without rapamycin. BMDMs that were treated with MHY1485 showed significantly increased expression of p-S6, NOS2, and FABP4, which was inhibited by rapamycin (Fig. 4f, g). These data suggest that activation of the mTORC1 pathway regulates M1 polarization in BMDMs and upregulates FABP4 expression.

Further analysis was performed to examine whether the mTORC1 pathway regulates M1-polarized macrophages to secrete FABP4 in vivo. Mice with myeloid lineage-specific mTORC1 activation were used as a model to investigate the role of M1-polarized macrophages.^13,24^ To determine whether mTORC1 activation contributes to macrophage polarization in the synovium, we generated mice (TSC1KO) with conditional ablation of the tuberous sclerosis complex 1 (TSC1) gene in myeloid cells using a Cre expression cassette under the control of the lysozyme proximal promoter.^25^ Thus, the target gene was specifically ablated in macrophages and neutrophils. Notably, we found that the expression of p-S6, NOS2 and FABP4 in the BMDMs of TSC1KO mice was significantly upregulated compared to that in controls (Fig. 4h). Synovial macrophages exhibited increased activation of mTORC1 (Fig. 4i), as well as significant increases in M1-like polarization and FABP4 expression (Fig. 4j) in the synovium in TSC1KO mice after AIA modeling compared to their littermate controls. HE staining showed obvious synovial hyperplasia, increased cellular infiltration, and higher synovitis scores in TSC1KO mice subjected to AIA than in control mice (Fig. S7A). In addition, an increase in synovial fibroblasts, enhanced invasion of FLSs (Fig. S7B, C), and elevated angiogenesis of H-type vessels (Fig. S7D) in the synovium, as well as increased OARSI scores (Fig. S7E), suppressed Col2a1 expression (Fig. S7F, G), and upregulated MMP13 expression (Fig. S7H) in cartilage, were observed in TSC1KO mice after AIA compared to the controls. A noticeable increase in osteoclasts was observed in TSC1KO mice 8 weeks after AIA modeling compared to the controls, but there was no significant difference in osteoclasts between TSC1KO mice and controls at 4 weeks after AIA modeling (Fig. S7I). These results demonstrated that M1-polarized macrophages secreted FABP4, which was triggered by activation of the mTORC1 pathway, to exacerbate RA progression.

### Reduced FABP4 secretion by M1-polarized macrophages prevents RA development by inhibiting macrophage mTORC1 activity

**Fig. 5:**
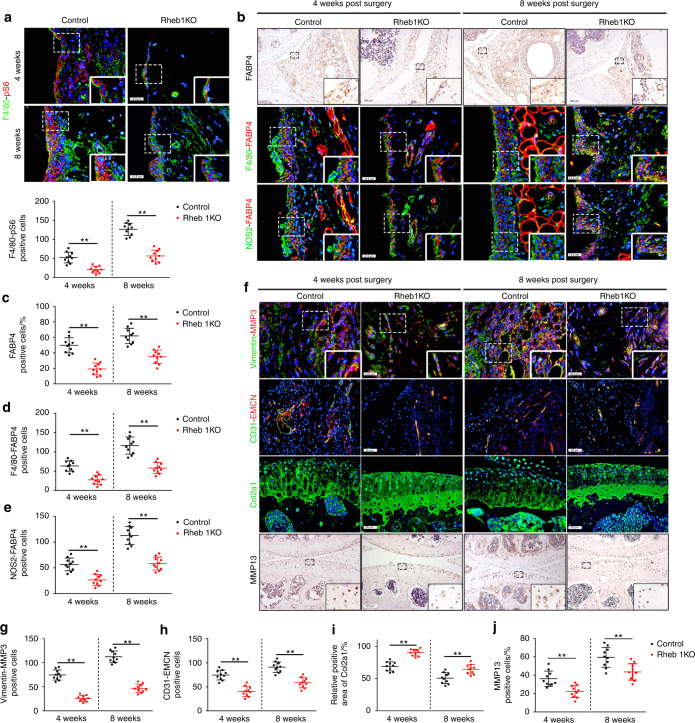
Inhibition of the mTORC1 pathway reduces FABP4 secretion by M1-polarized macrophages to attenuate RA progression. **a** Representative images and quantification of F4/80 and pS6 coimmunostaining in the synovium of controls and Rheb1KO mice at 4 and 8 weeks after AIA modeling (*n* = 10 per group). Scale bar: 12.5 µm. **b**–**e** Representative images (**b**) and quantitative analysis of FABP4 immunohistochemical staining (**c**) and coimmunostaining of FABP4 with F4/80 (**d**) or NOS2 (**e**) in the synovium of controls and Rheb1KO mice at 4 and 8 weeks after AIA modeling (*n* = 10 per group). Scale bars: 12.5 µm and 100 µm. **f**–**j** Representative images (**f**) and quantitative analysis of Vimentin and MMP3 coimmunostaining (**g**), CD31 and EMCN coimmunostaining (**h**), Col2a1 immunofluorescence staining (**i**), and MMP13 immunohistochemical staining (**j**) in the knee joints of controls and Rheb1KO mice at 4 and 8 weeks after AIA modeling (*n* = 10 per group). Scale bar: 12.5 µm, 25 µm, and 100 µm. Student’s *t*-test. ^**^P < 0.01. The data are shown as the mean ± SEM

To determine whether the inhibition of macrophage mTORC1 activity alleviates RA progression, Rheb1KO mice were generated, which had conditional ablation of the ras homolog enriched in the brain (Rheb1) gene, an upstream activator of mTORC1, in myeloid cells. In these mice, M2 polarization was enhanced, whereas M1 polarization was reduced, as previously reported.^25,26^ Suppression of mTORC1 activity was observed in Rheb1KO mice 4 and 8 weeks after modeling compared to the littermate controls (Fig. 5a). Notably, a significant reduction in FABP4 expression in M1-polarized macrophages was observed in Rheb1KO mice compared to controls (Fig. 5b–e). HE staining showed that Rheb1KO mice exhibited lower reduced of synovial hyperplasia, reduced cell infiltration, and significantly decreased synovitis scores after AIA modeling compared to control mice (Fig. S8A). Moreover, fewer synovial fibroblasts, weakened FLS invasion (Fig. 5f, g and Fig. S8B), and inhibited angiogenesis of H-type vessels (Fig. 5f, h) in the synovium, as well as lower OARSI scores (Fig. S8C), reduced Col2a1 degradation (Fig. 5f, i and Fig. S8D), and decreased MMP13 expression (Fig. 5f, j) in cartilage, were observed in Rheb1KO mice after AIA surgery compared to control mice. A significant decrease in osteoclasts was observed in Rheb1KO mice 8 weeks after AIA modeling compared to control mice, whereas the number of osteoclasts in Rheb1KO mice and controls was not significantly different at 4 weeks after AIA modeling (Fig. S8E). These data indicate that inhibiting macrophage mTORC1 activity to decrease FABP4 secretion by M1-polarized macrophages prevents the development of RA.

### BMS309403 and anagliptin decrease FABP4 levels in the synovium and serum to prevent RA progression

**Fig. 6:**
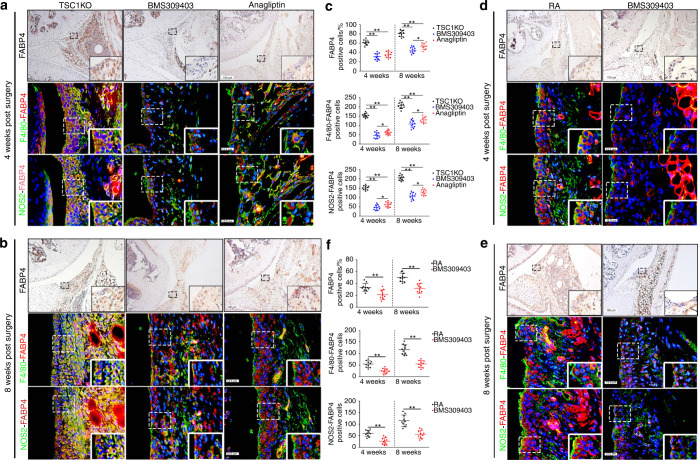
BMS309403 and anagliptin reduce FABP4 expression in murine synovial macrophages. **a**–**c** Representative images and quantification of FABP4 immunohistochemical staining and coimmunostaining of FABP4 with F4/80 or NOS2 in the synovium of TSC1KO mice treated with vehicle, BMS309403 or anagliptin for 4 and 8 weeks after AIA modeling (*n* = 10 per group). Scale bar: 12.5 µm and 100 µm. **d**–**f** Representative images and quantification of FABP4 immunohistochemical staining and coimmunostaining of FABP4 and F4/80 or NOS2 in the synovium of RA mice treated with vehicle or BMS309403 for 4 and 8 weeks (*n* = 10 per group). Scale bar: 12.5 µm and 100 µm. Student’s *t*-test or one-way ANOVA and Tukey’s multiple comparison test. ^*^P < 0.05, ^**^P < 0.01. The data are shown as the mean ± SEM

**Fig. 7:**
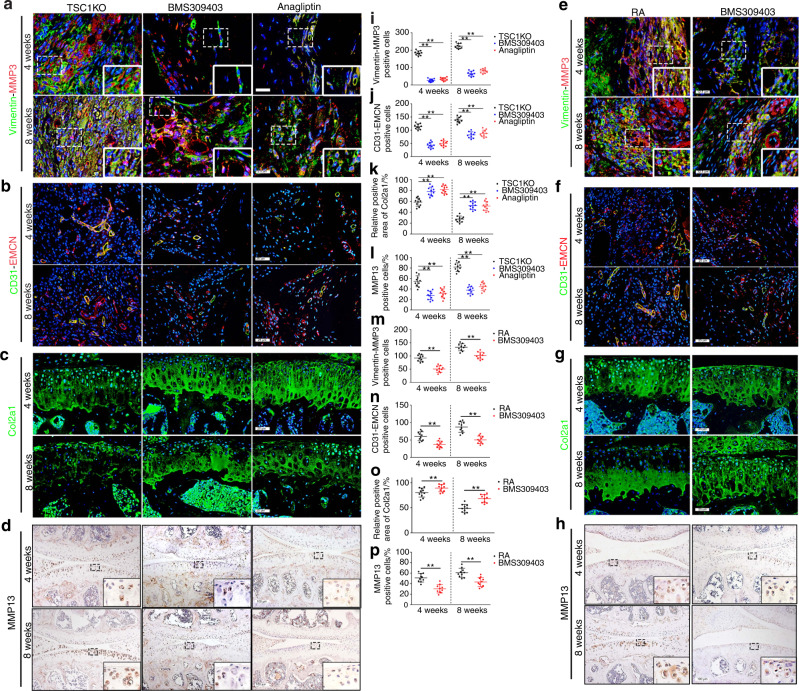
Inhibiting FABP4 prevents RA development. **a**–**d** Representative images of Vimentin and MMP3 coimmunostaining (**a**), CD31 and EMCN coimmunostaining (**b**), Col2a1 immunofluorescence staining (**c**), and immunohistochemical staining of MMP13 (**D**) in the knee joints of TSC1KO mice treated with vehicle, BMS309403 or anagliptin for 4 and 8 weeks after AIA modeling. Scale bar: 12.5 µm, 25 µm, and 100 µm. **e**–**h** Representative images of Vimentin and MMP3 coimmunostaining (**e**), CD31 and EMCN coimmunostaining (**f**), Col2a1 immunofluorescence staining (**g**), and immunohistochemical staining of MMP13 (**h**) in the knee joints of RA mice treated with vehicle or BMS309403 for 4 and 8 weeks. Scale bar: 12.5 µm, 25 µm, and 100 µm. **i**–**l** Quantification of Vimentin-MMP3 (**i**), CD31-EMCN (**j**), Col2a1 (**k**), and MMP13 (**l**) in TSC1KO mice treated with vehicle, BMS309403, or anagliptin for 4 and 8 weeks after AIA surgery (*n* = 10 per group). **m**–**p** Quantification of Vimentin-MMP3 (**m**), CD31-EMCN (**n**), Col2a1 (**o**), and MMP13 (**p**) in RA mice treated with vehicle or BMS309403 for 4 and 8 weeks (*n* = 10 per group). Student’s *t*-test or one-way ANOVA and Tukey’s multiple comparison test were used. ^**^P < 0.01. The data are shown as the mean ± SEM

Because high FABP4 expression was observed in synovial M1-polarized macrophages in RA patients and the RA mouse model, BMS309403 and anagliptin (dipeptidyl peptidase 4 inhibitors) were administered to TSC1KO mice for 4 and 8 weeks to inhibit endogenous FABP4 after AIA surgery. To further identify the therapeutic effects of FABP4 inhibitors on the development of the RA phenotype in vivo, we examined the histopathological characteristics of TSC1KO mice that were treated with BMS309403 or anagliptin after AIA surgery. Both BMS309403 and anagliptin significantly reduced the expression of FABP4 in the synovial M1-polarized macrophages (Fig. 6a–c) and serum (Fig. S2M) of TSC1KO mice. Excitingly, treatment with BMS309403 and anagliptin effectively prevented experimental RA progression by inhibiting synovitis (Fig. S9A), reducing synovial fibroblasts, weakening the invasive capacity of FLSs (Fig. 7a, i and Fig. S9B), and suppressing the angiogenesis of H-type vessels (Fig. 7b, j) in the synovium of TSC1KO mice compared to mice in the vehicle group. In addition, decreased OARSI scores (Fig. S9C), reduced Col2a1 degradation (Fig. 7c, k and Fig. S9D), and downregulated MMP13 expression (Fig. 7d, l) were observed in TSC1KO mice that were treated with BMS309403 or anagliptin compared to TSC1KO mice that were treated with vehicle. A decrease in osteoclasts was observed in TSC1KO mice that were treated with BMS309403 or anagliptin compared to mice in the vehicle group (Fig. S9E). Consistently, BMS309403 clearly decreased FABP4 expression in synovial M1-polarized macrophages (Fig. 6d–f) and serum (Fig. S2C) of C57BL/6 J mice after AIA modeling, which suppressed synovitis (Fig. S9F), the proliferation and invasion of FLSs (Fig. 7e, m and Fig. S9G), the angiogenesis of H-type vessels (Fig. 7f, n), knee joint cartilage thinness (Fig. S9H), Col2a1 degradation (Fig. 7g, o and Fig. S9I), and MMP13 expression (Fig. 7h, p) to alleviate experimental RA progression. A significant decrease in osteoclasts was observed in RA mice that were treated with BMS309403 for 8 weeks compared to mice in the vehicle group, whereas there was no significant difference in osteoclasts between the BMS309403 group and the vehicle group at 4 weeks after AIA modeling (Fig. S9J). These data indicate that BMS309403 and anagliptin inhibit the expression of FABP4 in M1-polarized macrophages to alleviate synovitis, angiogenesis and cartilage degeneration in RA.

## Discussion

Recent studies have demonstrated an essential role for synovial macrophages as important inflammatory effector cells during the pathogenesis of RA.^27,28^ The main morphological characteristic of synovitis is synovial hyperplasia, which is increased macrophage accumulation in the intimal lining of the synovium and is positively correlated with RA activity.^29,30^ Earlier studies have reported the accumulation and polarization of synovial macrophages in the articular cavity during RA development, suggesting a correlation between macrophages and RA.^31^ Activated M1 macrophages secrete a substantial amount of cytokines (such as IL-1β, IL-6, and TNF-α), thereby participating in the onset and progression of RA.^32^ However, the underlying mechanisms of RA remain unknown. An increasing number of studies have demonstrated a critical role of FABP4 secretion by macrophages in diabetes, hepatocellular carcinoma, and autoimmune disease via the regulation of inflammation and angiogenesis.^10,33,34^ In addition to IL-1β, IL-6, and TNFα, we found that FABP4 secretion by M1-polarized macrophages played a vital role in RA progression. Our findings demonstrate that FABP4, which is the main effector cytokine secreted by M1-polarized macrophages, was upregulated in the synovial tissue, cartilage and serum of patients and mice with RA. FABP4 was shown to promote macrophage proliferation and M1 polarization, enhance HUVEC tube formation, promote the proliferation, migration, invasion, and release of inflammatory cytokines by HUVECs and FLSs, and disrupt chondrocyte homeostasis. The upregulation of FABP4 promoted synovitis, angiogenesis, and cartilage degeneration to exacerbate the severity of experimental RA. The enhancement of mTORC1 activation elevated the expression of FABP4 in M1-polarized macrophages to exacerbate experimental RA progression. Importantly, inhibiting mTORC1 activation or FABP4 significantly mitigated the development of RA.

It has been well established that the mTORC1 pathway plays an important role in RA progression by regulating synovial hyperplasia, inflammation, and angiogenesis.^35,36^ Although inhibition of mTOR has a moderate effect on relieving arthritis in RA patients, this strategy cannot completely cure RA.^22^ Therefore, it is critical to elucidate the roles and mechanisms of specific cytokines regulated by the mTORC1 pathway in RA progression. Our findings demonstrated that mTORC1 activation induced macrophage polarization toward the M1 subtype and the secretion of FABP4 to promote RA development in mice. In contrast, the suppression of mTORC1 activation inhibited FABP4 expression in macrophages to attenuate RA progression. Consistently, the protein expression of FABP4, p-S6 and NOS2 was increased in macrophages induced with LPS or MHY1485, whereas rapamycin significantly reversed this increase in expression. The regulation of FABP4 by the mTORC1 pathway was also observed in hemangiomas.^23^ The abovementioned data showed that the upregulation of FABP4 secretion by M1-polarized macrophages was triggered by activation of the mTORC1 pathway.

Synovitis is the principal pathological characteristic of RA, and inflammatory cytokines are primarily secreted by macrophages to exacerbate RA progression.^37,38^ In addition to the inflammatory cytokines secreted by macrophages, we found that the upregulated secretion of FABP4 by M1-polarized macrophages enhanced the production of inflammatory cytokines (such as IL-1β, IL-6, and IL-18) in HUVECs and FLSs partially by activating the NF-κB pathway. Previous studies have demonstrated a proinflammatory role of FABP4 in allergic asthma and hyperuricemic nephropathy.^39,40^ Consistent with these findings, our study showed that rmFABP4 exacerbated synovitis in the knee joint of an RA mouse model.

Another important pathological feature of RA is angiogenesis, which is critical for synovial cell proliferation, migration, and invasion.^41–43^ Our results provide experimental evidence that FABP4 plays a vital role in angiogenesis within the RA synovium. In RA mice, rmFABP4 increased H-type vessel formation, which indicated an increase in the formation of new blood vessels.^21^ Furthermore, recombinant FABP4 promoted HUVEC tube formation by activating VEGFα protein expression. Exogenous FABP4 enhanced tube formation by endothelial cells, which was also observed in bronchopulmonary dysplasia and ovarian cancer.^44,45^ We suggest that FABP4-mediated angiogenesis exacerbates experimental RA progression. In addition to pannus formation, there are numerous invasive FLSs and M1-polarized macrophages in hyperplastic synovial tissue, which cause synovial infiltration during the development of RA. Recent studies have demonstrated that exogenous FABP4 promotes cell proliferation, migration, and invasiveness in colorectal cancer and breast cancer.^46,47^ Similar to these findings, we also found that recombinant FABP4 could promote the proliferation and invasion of FLSs in an RA mouse model, which suggested that FABP4 enhanced the aggressive phenotype of FLSs to promote RA development. Recombinant FABP4 also enhanced the proliferation and M1 polarization of macrophages in vivo and in vitro. Moreover, recombinant FABP4 promoted the proliferation, migration, and invasiveness of HUVECs and FLSs in vitro. These findings provide evidence for synovial infiltration in the knee joint during the progression of RA. Our study elucidates the essential role of FABP4 in macrophages, HUVECs and FLSs during the pathogenesis of RA and supports the underlying mechanism of RA pathophysiology.

Cartilage destruction gradually occurs and is triggered by persistent synovitis, synovial hyperplasia, infiltration, and pannus formation during RA progression.^48^ Zhang et al. previously reported that knockout or pharmaceutical inhibition of FABP4 could significantly alleviate cartilage degradation, osteophyte formation and subchondral bone sclerosis in OA induced by a high-fat diet in mice, but it had no significant effect on lean mice that were fed a standard diet.^49^ However, Zhang et al. did not examine the source of FABP4 or the mechanism of FABP4 in chondrocytes in OA. Whether FABP4 regulates articular cartilage degradation in RA is still unclear. Our study demonstrated that FABP4 was involved in cartilage degradation mediated by the synovium. Worsened articular cartilage degeneration was observed in RA mice treated with rmFABP4. Furthermore, recombinant FABP4 enhanced catabolism and suppressed anabolism in primary chondrocytes partly by activating the NF-κB pathway. Interestingly, the upregulation of FABP4 was also observed in the articular cartilage of RA mice. Since a previous study showed that IL-1β could stimulate the expression of FABP4 in ATDC5 cells (a chondrogenic cell line),^50^ we hypothesized that FABP4 upregulation in RA chondrocytes was induced by excessive proinflammatory factors (IL-1β, IL-6 and TNF-α) derived from synovial M1 macrophages, FLSs and ECs. These data demonstrated that FABP4 secretion by synovial macrophages exacerbated cartilage degeneration by disrupting chondrocyte homeostasis through activation of the NF-κB pathway in RA.

Importantly, our findings were of clinical relevance and showed that BMS309403 (a FABP4 inhibitor) could effectively alleviate the development of RA by ameliorating synovitis, angiogenesis, and cartilage degradation in mice. It has been reported that BMS309403 significantly inhibits the proliferation, migration, and tube formation induced by exogenous FABP4.^51,52^ Based on further studies that evaluated the effects of BMS309403 in vitro, we found that BMS309403 significantly reversed the phenotypes induced by recombinant FABP4 and M1-polarized macrophage supernatant. Namely, BMS309403 attenuated HUVEC tube formation, inhibited the proliferation, migration, invasion, and production of inflammatory cytokines in HUVECs and FLSs, and restored the imbalance in chondrocyte metabolism, which might be associated with RA pathogenesis. These data demonstrated that FABP4 served as the main effector cytokine of M1-polarized macrophages that affects HUVECs, FLSs, and chondrocytes. Interestingly, we found that anagliptin (a dipeptidyl peptidase 4 inhibitor) also decreased the level of FABP4 in serum and synovial macrophages in an RA mouse model,^53^ which alleviated the severity of RA. Taken together, these results suggest the potential clinical value of using a FABP4 inhibitor to treat RA.

Although we have demonstrated the critical role of FABP4 in RA, several limitations have yet to be overcome. First, we demonstrated that FABP4 was an important cytokine that promoted synovitis, angiogenesis, and cartilage degradation, thus accelerating the progression of RA. However, FABP4 inhibition could only partially reverse RA progression, which indicated that other cytokines secreted by macrophages (such as IL-1β and TNFα) were factors in the pathogenesis of RA. Second, our study revealed that BMS309403, a FABP4 inhibitor, was an effective drug to treat experimental RA in the RA mouse model. However, to realize clinical translation, the treatment effects and side effects of this drug merit further analysis. Third, an obvious increase in osteoclasts was observed in RA mice that were treated with rmFABP4, and a significant decrease in osteoclasts was observed in RA mice that were administered BMS309403, which indicates that FABP4 might play a role in regulating osteoclast activity, but the specific mechanism needs to be further assessed. Finally, further studies are needed to explore the potential mechanism of macrophage secretion and the molecular interaction of FABP4. It is necessary to investigate whether there are cell surface molecules that can interact with FABP4 to transmit its signal and which complexes or ligands inside or outside the cell can act as nodes for the signal transduction of these molecules.

In conclusion, our findings indicate that the upregulation of FABP4, a key effector cytokine in M1-polarized macrophages, promoted the proliferation, migration, invasiveness, and production of inflammatory cytokines in HUVECs and FLSs, enhanced HUVEC tube formation, and disrupted chondrocyte homeostasis (Fig. S10). Additionally, BMS309403 effectively inhibited the effect of FABP4 on HUVECs, FLSs, and chondrocytes. Furthermore, FABP4 promoted synovitis, angiogenesis, and cartilage degeneration to exacerbate the severity of RA, while BMS309403 and anagliptin effectively inhibited the pathological effects of FABP4 on RA progression. Hence, FABP4 represents a potential therapeutic target for RA treatment.

## Methods

### Human samples

Control human synovial tissue was obtained from the individuals with anterior cruciate ligament injury with no history of arthritic diseases (*n* = 16). Human RA synovium and cartilage were obtained from patients who underwent total knee replacement surgery (*n* = 16). Control cartilage was obtained from the lateral tibial plateau with reduced cartilage destruction in OA patients who underwent total knee replacement surgery (*n* = 16). The clinical characteristics of RA patients are listed in Table S1. RA and control adipose tissue were obtained from the infrapatellar fat pads (IPFPs) of RA and OA patients who underwent total knee replacement surgery (*n* = 6 per group). Control adipose tissue was obtained from the infrapatellar fat pads (IPFPs) of RA patients who underwent total knee replacement surgery (*n* = 6). Human synovial tissue was analyzed by hematoxylin and eosin (HE) staining, immunofluorescence (IF) staining, and immunohistochemistry (IHC). All human samples were obtained from the Third Affiliated Hospital of Southern Medical University (Guangzhou, China). All patients provided informed consent to use their clinical information for scientific research. This study was approved by the Ethics Committee of the Third Affiliated Hospital of Southern Medical University.

### Animals

Lys-MCre mice and Tsc1^flox/flox^ mice were purchased from Jackson Laboratory (Bar Harbor, ME, USA; Jax nos. 004781 and 005680, respectively). Rheb1^flox/flox^ mice were a generous gift from Professor Bo Xiao of Sichuan University. To generate myeloid lineage-specific *Tsc1-* or *Rheb1-*knockout mice, Lys-MCre mice were crossed with Tsc1^flox/flox^ mice and Rheb1^flox/flox^ mice. Mice with myeloid lineage-specific deletion of *Tsc1* were called TSC1KO mice, and those with *Rheb1* deletion were called Rheb1KO mice. Littermates carrying Tsc1^flox/flox^ or Rheb1^flox/flox^ without Cre were used as wild-type mice. Routine genotyping of tail DNA was performed according to the Jackson Laboratory’s instructions. The gross appearance of TSC1KO mice and Rheb1KO mice has previously been reported.^25^ One hundred and twenty eight-week-old male C57BL/6 J mice were purchased from the Experimental Animal Centre of Southern Medical University (Guangzhou, China). To examine the role of FABP4 in experimental RA, eighty twelve-week-old male C57BL/6 J mice were randomly separated into three groups: the sham group was intra-articularly treated with phosphate-buffered saline (*n* = 30); the antigen-induced arthritis (AIA) group was intra-articularly injected with phosphate-buffered saline (*n* = 30); and the rmFABP4 group was administered rmFABP4 after AIA modeling (*n* = 20). To determine the role of the mTORC1 pathway in experimental RA, twenty twelve-week-old male TSC1KO mice, twenty twelve-week-old male Rheb1KO mice, and their littermates were subjected to AIA surgery. To examine the role of FABP4 inhibition in experimental RA, sixty twelve-week-old male TSC1KO mice were randomly separated into three groups: Group 1 (*n* = 20) animals underwent AIA and were administered physiological saline; Group 2 (*n* = 20) animals underwent AIA with administration of BMS309403; and Group 3 (*n* = 20) animals underwent AIA and were administered anagliptin. Additionally, forty twelve-week-old male C57BL/6 J mice were randomly separated into two groups: Group 1 (*n* = 20) animals underwent AIA and were treated with physiological saline; and Group 2 (*n* = 20) animals underwent AIA with treatment of BMS309403. The animals were sacrificed at 4, 8 or 12 weeks after AIA modeling, and 10 mice were sacrificed at each time point. All animals were provided a standard diet and were housed in pathogen-free cages (5 mice per cage) with constant temperature and humidity. The circadian rhythm was maintained at 12 h. Euthanasia was performed by an overdose of ketamine/xylazine followed by cervical dislocation. All animal experiments were approved by the Southern Medical University Committee Animal Care and Use Committee and were performed in accordance with the Committee’s guidelines and regulations.

### Antigen-induced arthritis (AIA)

The experimental mouse model of antigen-induced arthritis (AIA) was established as described previously.^54^ Briefly, 12-week-old male C57BL/6 J mice, TSC1KO mice and Rheb1KO mice were injected subcutaneously on Day 0 with 500 μg of methylated bovine serum albumin (mBSA; Sigma–Aldrich, USA) in 50 μL of phosphate buffered saline (PBS) emulsified in 50 μL of Freund’s complete adjuvant (CFA; Sigma–Aldrich, USA). Two weeks later, 10 μg of mBSA (in 10 μL sterile saline) was injected into the right knee joint of each mouse. Mice that were intra-articularly injected with PBS were used as controls. On Day 3 after AIA, the mice were administered rmFABP4, BMS309403, anagliptin or vehicle.

### Histological analysis

Total knee joints were fixed in 4% paraformaldehyde for 24 h, decalcified with 0.5 mol·L^−1^ EDTA (pH 7.4) for 21 days and subsequently embedded in paraffin. The samples were cut into 4 µm-thick sections for hematoxylin and eosin (HE) and Safranin O/Fast Green staining. The synovial membranes in routine HE-stained slides were graded according to three synovial membrane features (synovial lining cell layer, stromal cell density and inflammatory infiltrates). The alterations were ranked on a scale: none (score: 0), slight (score: 1), moderate (score: 2), and strong (score: 3).^55^ Cartilage degeneration in safranin O/fast green-stained sections was graded using the Osteoarthritis Research Society International (OARSI) scoring system. We applied this 0–6 subjective scoring system to all four quadrants of the joint: medial femoral condyle (MFC), medial tibial plateau (MTP), lateral femoral condyle (LFC), and lateral tibial plateau (LTP). A score of 0 represents normal cartilage, 0.5 = loss of proteoglycan with an intact surface, 1 = superficial fibrillation without loss of cartilage, 2 = vertical clefts and loss of surface lamina, 3 = vertical clefts/erosion to the calcified layer lesion over 1%–25% of the quadrant width, 4 = lesion reaches the calcified cartilage over 25%–50% of the quadrant width, 5 = lesion reaches the calcified cartilage over 50%–75% of the quadrant width, and 6 = lesion reaches the calcified cartilage over >75% of the quadrant width. Each section was assessed by two blinded, independent graders, and the average score was used for statistical analysis.

### Cell preparation

Human umbilical vein endothelial cells (HUVECs) and fibroblast-like synoviocytes (FLSs) were purchased from the ATCC. Human primary chondrocytes were obtained from the tibial plateaus of OA and RA patients. Murine primary chondrocytes were obtained from the tibia cartilage of newborn mice as previously described.^56^ Bone marrow-derived macrophages (BMDMs) were obtained from the bone marrow of 6-week-old female C57BL/6 J and TSC1KO mice and their littermate controls. Macrophages and their supernatant were collected after being stimulated with 50, 200, or 500 ng·mL^−1^ LPS (Invitrogen, San Diego, CA, USA) for 12 h or 24 h. HUVECs, FLSs, and primary chondrocytes were treated with 0.4 μmol·L^−1^ recombinant human FABP4 (#RPB693Hu01, Cloud-Clone Corp., China), M1-polarized macrophage supernatant, or 20 μmol·L^−1^ BMS309403 (MedChemExpress, Shanghai, China) for 1 hour to examine pathway activation or 24 h to examine phenotypic alterations. HUVECs were treated with rhFABP4 for 24 h after 24 h of FABP lentivirus infection. Macrophages were collected after the administration of 500 ng·mL^−1^ LPS, 50 μmol·L^−1^ MHY1485 (MedChemExpress, Shanghai, China), or 100 μmol·L^−1^ rapamycin (MedChemExpress, Shanghai, China) for 6 h.

### Knockdown of FABP4 in HUVECs

The shRNAs targeting human FABP4 were carried by the PLKO.1 lentivirus packaging system provided by Hanbio Biotechnology (Shanghai, China). Lentiviruses carrying three shRNAs (FABP4-shRNA1: GGCATGGCCAAACCTAAA-TGATCA; FABP4-shRNA2: GGGTGTCCTGGTACA-TGTGCAGAAA; FABP4-shRNA3: CCACGAGAGTTTATGAGAGAGCATA) targeting FABP4 and the corresponding negative control (FABP4-control: TTCTCCGAACGTGTCACGTAA) were used to knock down FABP4 in HUVECs. Briefly, cells at 40%–50% confluence were infected with lentivirus supernatant and incubated at 37 °C for 48 h. The knockdown efficiency of shRNAs targeting FABP4 was determined by Western blotting.

### Tube formation assay

HUVECs were seeded with 200 µL of Matrigel (BD Biosciences, NSW, Australia) in a 24-well dish at a cell density of 65 000 cells per well. Tube formation was assessed after 12 h by visual microscopy using an inverted microscope (Olympus, Tokyo, Japan). The different parameters of tube formation were analyzed using the Angiogenesis analysis plugin in ImageJ software.

### Proliferation assay

The proliferation rates of HUVECs and FLSs were measured using a CCK-8 assay (Dojindo Laboratories, Tokyo, Japan). The absorbance was measured at 450 nmol·L^−1^ with a microplate reader (BOP-TEK) according to previously published methods.^57,58^

HUVECs and FLSs were plated in a 12-well plate at a density of 150 000 cells per well. After being infected with rhFABP4, M1-polarized macrophage supernatant, or BMS309403 for 24 h, the cells were incubated with 100 μg·mL^−1^ BrdU for 2 h. For quantification, the number of BrdU-positive cells was determined as the average count of 3 random fields of view. Images were obtained using a fluorescence microscope (Olympus).

### Migration assay

HUVECs (30 000 cells per well) or FLSs (50 000 cells per well) were suspended in high glucose Dulbecco’s modified Eagle’s medium (DMEM) without fetal bovine serum (FBS) (Gibco, Gaithersburg, MD, USA), and 200 µL of this cell suspension was seeded into Transwell inserts (PC member, 6.5 mm diameter, 8 µm pores; Corning #3422). The inserts were placed in a 24-well plate containing 800 µL of DMEM with 10% FBS. The cells that migrated to the lower side of the Transwell insert were fixed in methanol and stained with 2% crystal violet. After being washed extensively with PBS to remove any excess crystal violet stain, the number of cells that had migrated was counted in three different representative high‐power fields per insert with an inverted microscope (Olympus).

### Invasion assay

HUVECs (150 000 cells per well) or FLSs (200 000 cells per well) were suspended in high glucose DMEM without FBS (Gibco), and 200 µL of this cell suspension was seeded into Transwell inserts (PC member, 6.5 mm diameter, 8 µm pores; Corning #3422; Corning, Sunnyvale, CA, USA). The inserts were coated with 16% Matrigel (356231; BD Biosciences, San Diego, CA, USA) in DMEM without FBS. After 5 h, the inserts were placed in a 24-well plate containing 800 µL of DMEM containing 20% FBS. The cells that migrated to the lower side of the Transwell insert were fixed in methanol and stained with 2% crystal violet. After being washed extensively in PBS to remove excess crystal violet stain, the number of cells that had migrated was counted in three different representative high-power fields per insert using an inverted microscope (Olympus).

### Scratch assay

HUVECs or FLSs were seeded in six-well plates and grown to confluence. A scratch wound was made in each well using a sterile pipette tip. The cells were subsequently exposed to DMEM containing 10% FBS for 12 h, 24 h, or 36 h. HUVEC or FLS migration across the wound margins was assessed, photographed, and measured using ImageJ software. The average percentage of wound healing was calculated in three different fields at 12 h, 24 h, or 36 h.

### Flow cytometry

After being treated with vehicle, LPS, IL4, FABP4 or BMS309403, BMDMs were harvested and washed twice with cold PBS. For cell-surface analysis, the cells were stained with F4/80 (123119, BioLegend), CD11b (101227, BioLegend) and CD86 (105007, BioLegend) at the recommended antibody concentrations at 4 °C for 30 min after being incubated with FcRblock (101319, BioLegend) at 4 °C for 10 min. For CD206 (141707, BioLegend) staining, the cells were fixed and permeabilized before being incubated at 4 °C for 30 min. Cells were analyzed using an LSRFortessa (BD). Data were acquired and processed using Flow Jo software.

### Immunohistochemistry and immunofluorescence analysis

Tartrate-resistant acid phosphatase (TRAP) staining (Sigma–Aldrich, Missouri, USA) was performed according to the manufacturer’s instructions. All slides were prepared as described previously.^59^ After deparaffinization and rehydration, the sections were soaked in citrate buffer (10 mmol·L^−1^ citric acid, pH 6.0) overnight at 60 °C to unmask the antigen. For immunohistochemical staining, 3% hydrogen peroxide was added and incubated for 10 min to inactivate any endogenous peroxidase activity. The sections were blocked with 1% goat serum at 37 °C for 1 h. FABP4 (1:100 for IHC, Abclone #A0232) and MMP13 (1:100 for IHC, Proteintech #18165-1-AP) primary antibodies were added and incubated overnight at 4 °C. For immunohistochemical staining, the sections were stained with horseradish peroxidase-conjugated secondary antibodies (Jackson ImmunoResearch Laboratories, Inc., West Grove, PA, USA), 3,3′-diamino-benzidine (DAB; ZSGB-Bio, Beijing, China) was used to observe chromogens, and hematoxylin was used as a counterstain. For immunofluorescence analysis, the primary antibodies were against F4/80 (1:100 for IF, Santa Cruz #sc-377009; Santa Cruz Biotechnology, Santa Cruz, CA, USA), NOS2 (1:100 for IF, Santa Cruz #sc-7271), FABP4 (1:100 for IF, Abclone #A0232; ABclone, Woburn, MA, USA), vimentin (1:100 for IF, Santa Cruz #sc-6260), MMP3 (1:100 for IHC, Proteintech #17873-1-AP; Proteintech, Rosemont, IL, USA), CD31 (1:100 for IF, R&D #AF3628; R&D Biosystems, Minneapolis, MN, USA), EMCN (1:100 for IF, Santa Cruz #sc-65495), and Col2a1 (1:100 for IF, Abcam #ab34712). For secondary reactions, the sections were stained with species-matched Alexa 488 or Alexa 594 dye-labeled secondary antibodies (Life Technologies, Carlsbad, CA, USA). Nuclei were labeled with 4,6-diamidino-2-phenylindole (DAPI; Thermo Fisher Scientific, Waltham, MA, USA), and images were obtained using a fluorescence microscope (Olympus). Under high magnification, three fields of the medial synovium were selected, and the number of positively stained macrophages in the synovium was calculated to obtain a mean value. The sections were randomly coded, and three sections per joint were scored by two blinded observers.

### rmFABP4, BMS309403 and anagliptin treatment

rmFABP4 (#RPB693Mu01, Cloud-Clone Corp., China) (5 μg per 10 μL per week) was intra-articularly injected into C57BL/6 J mice with antigen-induced arthritis once per week for 4 or 8 weeks, while BMS309403 (5 mg·kg^−1^) was administered twice per week to C57BL/6 J and TSC1KO mice with antigen-induced arthritis via an intraperitoneal injection for 4 or 8 weeks. Anagliptin (0.3%) was administered to TSC1KO mice with antigen-induced arthritis for 4 and 8 weeks. The control groups were treated with saline for an equivalent time.

### Western blot analysis

Cells and synovial tissue were immediately lysed for 10 min on ice in lysis buffer (62.5 mmol·L^−1^ Tris-HCl, pH 6.8, 10% glycerol, 2% sodium dodecyl sulfate, 50 mmol·L^−1^ dithiothreitol, and 0.01% bromophenol blue) containing phosphatase and protease inhibitors. Cell lysates were analyzed by sodium dodecyl sulfate–polyacrylamide gel electrophoresis and transferred to a nitrocellulose membrane (Bio–Rad Corp., Hercules, CA, USA). The blots were probed with primary antibodies, and immunoreactive proteins were revealed using an enhanced chemiluminescence kit (Santa Cruz Biotechnology Inc.).

### ELISA

We used human and mouse FABP4 ELISA kits (Elabscience Biotechnology, Bethesda, MD, USA: #E-EL-H0285c and #E-EL-M2404c) to analyze the level of FABP4 in the supernatant of macrophages stimulated with lipopolysaccharide (LPS), the serum of C57BL/6 J and TSC1KO mice, and human serum and synovial fluid. ELISA was performed according to the manufacturer’s instructions.

### Statistical analysis

All experiments were performed in duplicate or triplicate and were observed by independent observers. Differences between two groups were analyzed using Student’s *t* test, while differences between the three groups were analyzed by one-way analysis of variance (ANOVA) and Tukey’s multiple comparison test. All statistical analyses were performed with GraphPad Prism 6.0 (GraphPad Software Inc., La Jolla, CA, USA). The results are presented as the mean ± standard error (SEM), and *P* < 0.05 was considered to be statistically significant.

## Supplementary information

Supplementary file

## Data Availability

All data generated or analyzed during this study are included in this submitted article and its additional files.
