# Correction: FABP4 secreted by M1-polarized macrophages promotes synovitis and angiogenesis to exacerbate rheumatoid arthritis

**DOI:** 10.1038/s41413-023-00271-y

**Published:** 2023-07-25

**Authors:** Dong Guo, Chuangxin Lin, Yuheng Lu, Hong Guan, Weizhong Qi, Hongbo Zhang, Yan Shao, Chun Zeng, Rongkai Zhang, Haiyan Zhang, Xiaochun Bai, Daozhang Cai

**Affiliations:** 1 Department of Joint Surgery, Center for Orthopedic Surgery, The Third Affiliated Hospital of Southern Medical University, Guangzhou, China; 2 Department of Orthopedics, Orthopedic Hospital of Guangdong Province, Academy of Orthopedics, Guangdong Province, The Third Affiliated Hospital of Southern Medical University, Guangzhou, China; 3 The Third School of Clinical Medicine, Southern Medical University, Guangzhou, China; 4 Guangdong Provincial Key Laboratory of Bone and Joint Degeneration Diseases, Guangzhou, China; 5 Department of Orthopedic Surgery, Shantou Central Hospital, Affiliated Shantou Hospital of Sun Yat-sen University, Shantou, China; 6 State Key Laboratory of Organ Failure Research, Department of Cell Biology, Southern Medical University School of Basic Medical Sciences, Guangzhou, China

**Keywords:** Bone, Pathogenesis

Correction to: *Bone Res* 10.1038/s41413-022-00211-2, published online 22 June 2022

During a re-read of our previously published article^1^ in *Bone Research*, we regrettably found that the use of statistical chart for NOS2-FABP4 positive cells on the right side in figure 1(d), the quantification of NOS2-FABP4 positive cells in figure 3(a), CD31-EMCN positive cells in supplementary figure 2(h) and branch point in supplementary figure 3(d) were inaccurate. Although those statistical charts do not affect the results and conclusion in the article, all the authors agree to correct this negligence by providing correctly drawn figure 1(d), figure 3(a), supplementary figure 2(h) and supplementary figure 3(d), which are presented below, to guarantee the accuracy of the statistics. We sincerely regret and apologize for any inconvenience caused.

The corrected figure 1 should read:
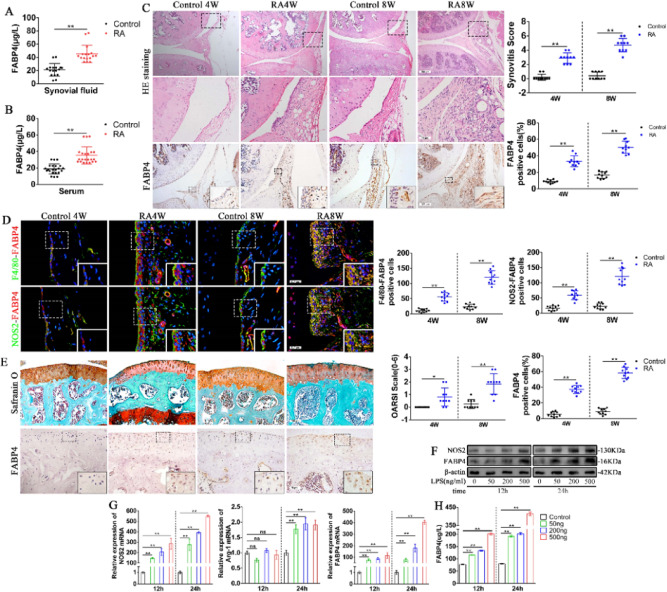


The corrected figure 3 should read:
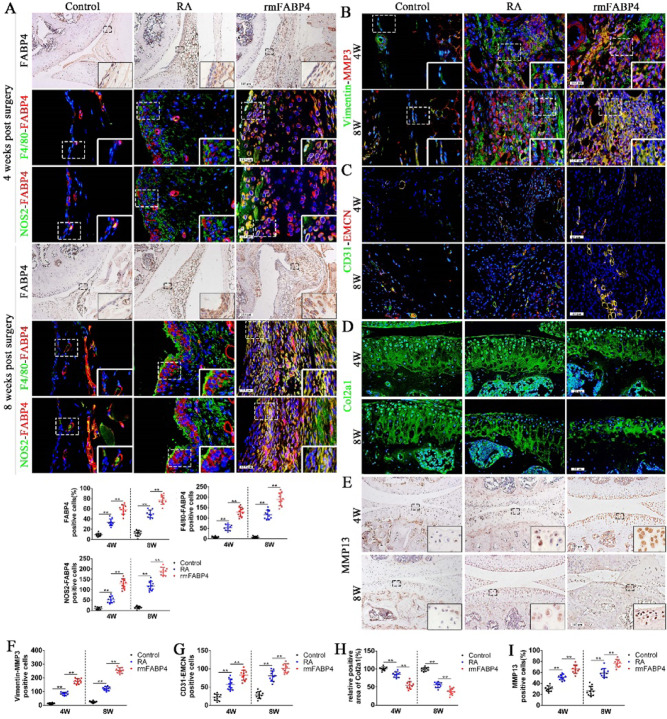


The corrected supplementary figure 2 should read:
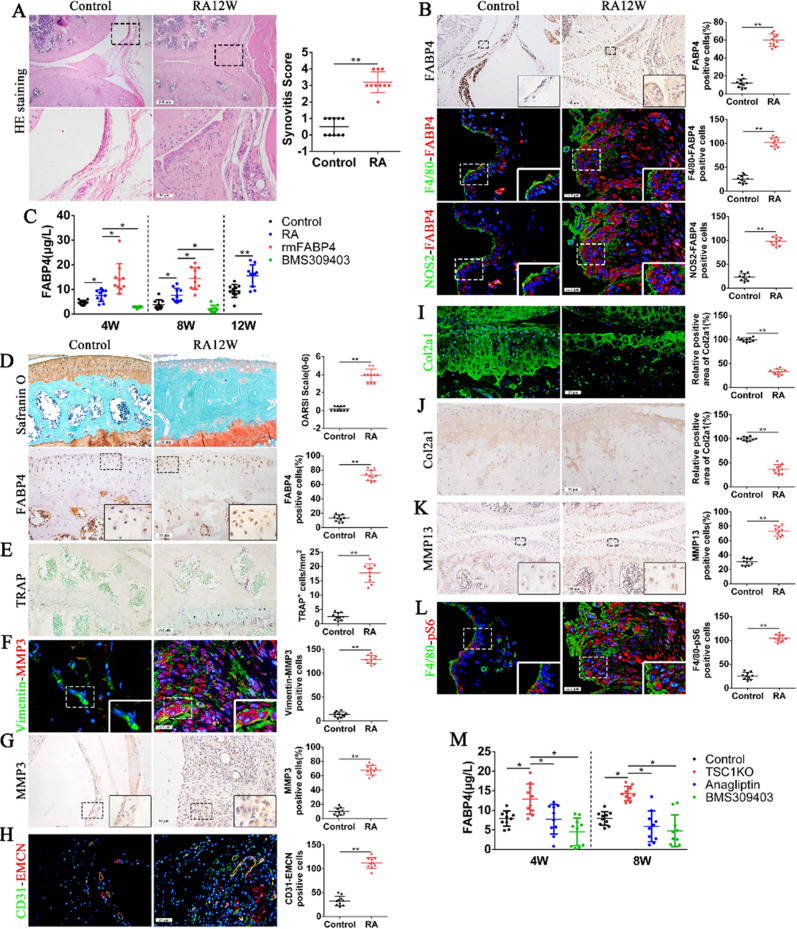


The corrected supplementary figure 3 should read:
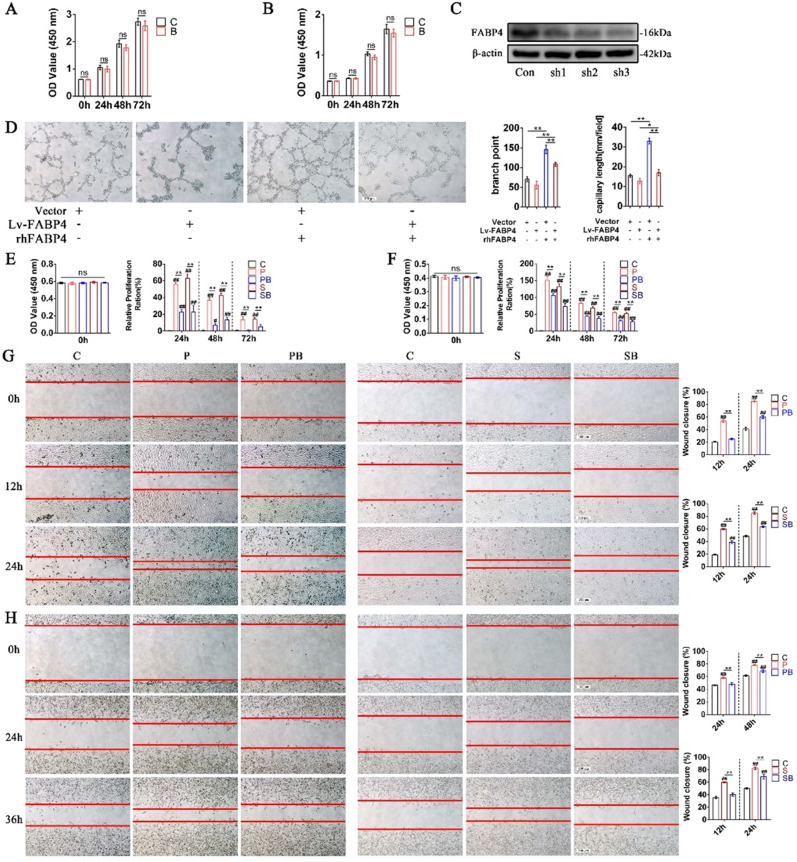


The original article^1^ was updated.
