# Supplementary material for: FABP4 secreted by M1-polarized macrophages promotes synovitis and angiogenesis to exacerbate rheumatoid arthritis
Source: Bone Res. 2022 Jun 22;10:45. doi: 10.1038/s41413-022-00211-2 (PMC9213409; doi:10.1038/s41413-022-00211-2)
Supplement: Supplementary file 1 — Supplementary file [file 41413_2022_211_MOESM1_ESM.docx]

**FABP4 secreted by M1-polarized macrophage promotes synovitis and angiogenesis to aggravate rheumatoid arthritis**

Dong Guo^1,2,3,4^, Chuangxin Lin^6^, Yuheng Lu^1,2,3,4^, Hong Guan^1,2,3,4^, Weizhong Qi^1,2,3,4^, Hongbo Zhang^1,2,3,4^, Yan Shao^1,2,3,4^, Chun Zeng^1,2,3,4^, Rongkai Zhang^1,2,3,4^, Haiyan Zhang^1,2,3,4^*, Xiaochun Bai^4,5^*, Daozhang Cai^1,2,3,4^*

Running title: FABP4 promotes synovitis and angiogenesis in RA.

**Supplementary Materials and Figures**

| **Characteristics** | **N Mean ± SD** | **Range or Percentage** |
| --- | --- | --- |
| Age | 61.94 ± 12.77 | 31-80 |
| Female | 10 | 62.50% |
| Male | 6 | 37.50% |
| ACPA positive | 9 | 56.25% |
| IgM-RF positive | 9 | 56.25% |
| IgG-RF positive | 10 | 62.50% |
| hs-CRP (mg/L) | 89.80 ± 58.89 | 1.33-205.50 |
| ESR (mm/h) | 58.44 ± 31.26 | 11-110 |
| DAS28-CRP | 4.97 ± 1.08 | 3.06-7.03 |

Table S1 Clinical characteristics of recruited RA patients (n = 16).

ACPA: anti-citrullinated protein antibody; IgM-RF: IgM rheumatoid factor; IgG-RF: IgG rheumatoid factor; hs-CRP: hypersensitive C-reactive protein; ESR: erythrocyte sedimentation rate; DAS28-CRP: 28-joint Disease Activity Score using C-reactive protein.


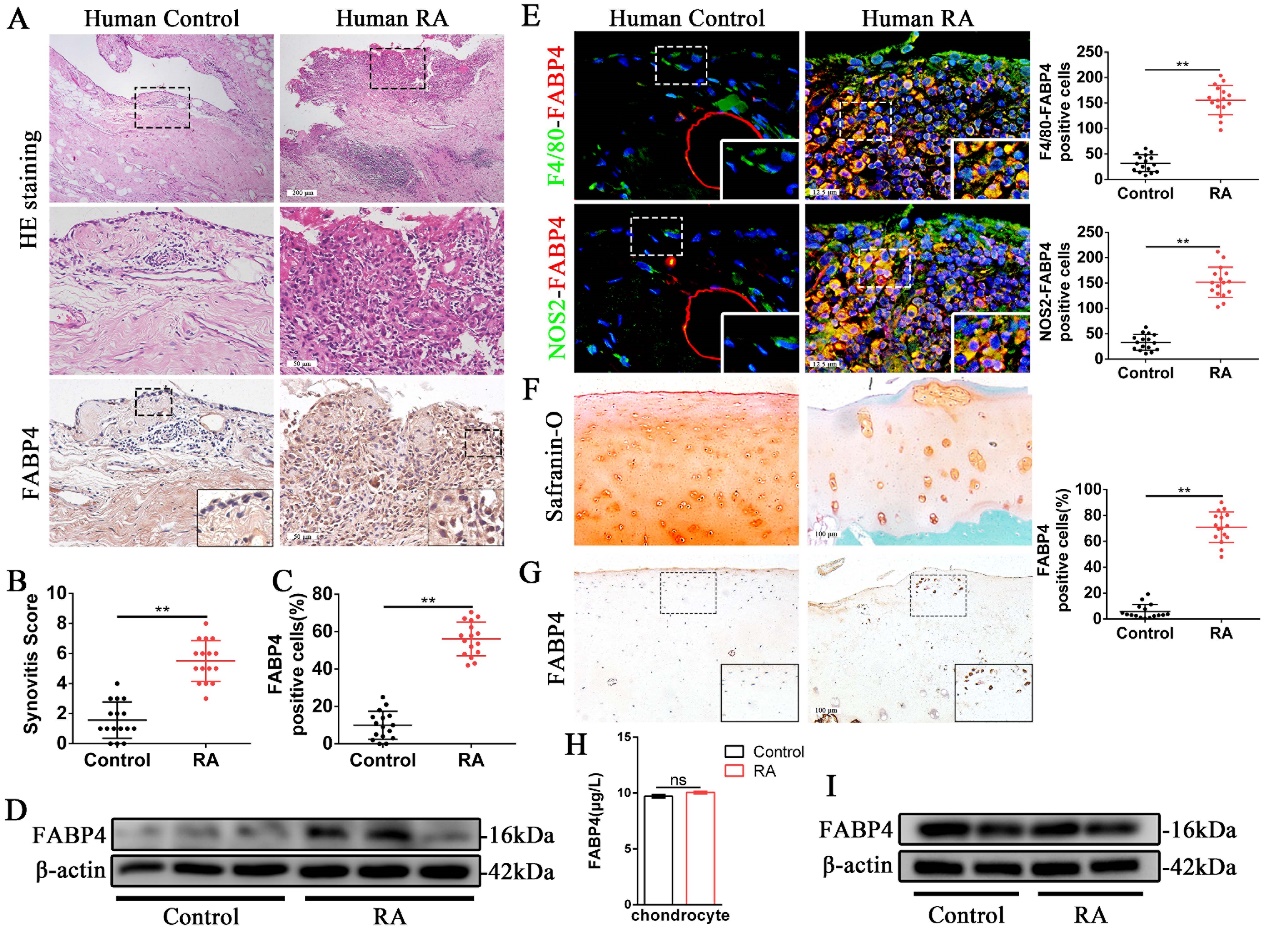


**Figure S1.** The expression of FABP4 in patients with RA. **(A)** Representative images of H&E staining and immunohistochemistry of FABP4 in control and RA human synovial tissue. Scale bars: 50 μm and 200 μm. **(B, C)** Quantification of the synovitis score (B) and FABP4 (C) in control and RA human synovial tissues (n = 16 per group). **(D)** Western blot of FABP4 expression in human synovial tissue. **(E)** Representative images and quantitative analysis of coimmunostaining of FABP4 with F4/80 or NOS2 in human synovium (n = 16 per group). Scale bar: 12.5 µm. **(F, G)** Representative images and quantification of Safranin O and Fast Green staining (F) and FABP4 immunohistochemical staining (G) in knee cartilage from control and RA patients (n = 16 per group). Scale bars: 50 μm and 100 μm. **(H)** FABP4 concentrations assessed by ELISA of the supernatant of chondrocytes from knee cartilage of control and RA patients (n = 3 per group). **(I)** Western blot of FABP4 expression in human adipose tissue. Student’s *t*-test. ^**^P < 0.01; ns, no significance. Data are shown as mean ± SEM.





**Figure S2.** Phenotypes of RA mice at 12 weeks and FABP4 concentration in the serum of C57BL/6 and TSC1KO mice. **(A, B)** Representative images and quantification of H&E staining (A), FABP4 immunohistochemical staining and coimmunostaining of FABP4 with F4/80 or NOS2 (B) in the knee joints of control and RA mice at 12 weeks after AIA modeling (n = 10 per group). Scale bar: 12.5 µm, 50 µm, 100 µm, and 200 µm. **(C)** FABP4 concentrations assessed by an ELISA in the serum of control and RA mice treated with a vehicle, rmFABP4 or BMS309403 (n = 10 per group). **(D-L)** Representative images and quantification of Safranin O and Fast Green staining, FABP4 immunohistochemical staining (D), TRAP staining (E), Vimentin and MMP3 coimmunostaining (F), MMP3 immunohistochemical staining (G), CD31 and EMCN coimmunostaining (H), Col2a1 immunofluorescence staining (I), Col2a1 immunohistochemical staining (J), MMP13 immunohistochemical staining of (K), and F4/80 and pS6 coimmunostaining (L) in the knee joints of control and RA mice at 12 weeks after AIA modeling (n = 10 per group). Scale bar: 12.5 µm, 50 µm, 100 µm, and 200 µm. **(M)** FABP4 concentrations assessed by an ELISA in the serum from controls and TSC1KO mice treated with a vehicle, BMS309403 or anagliptin for 4 and 8 weeks after AIA surgery (n = 10 per group). Student’s *t*-test or one-way ANOVA and Tukey’s multiple comparison test. ^*^P < 0.05, ^**^P < 0.01. Data are shown as the mean ± SEM.


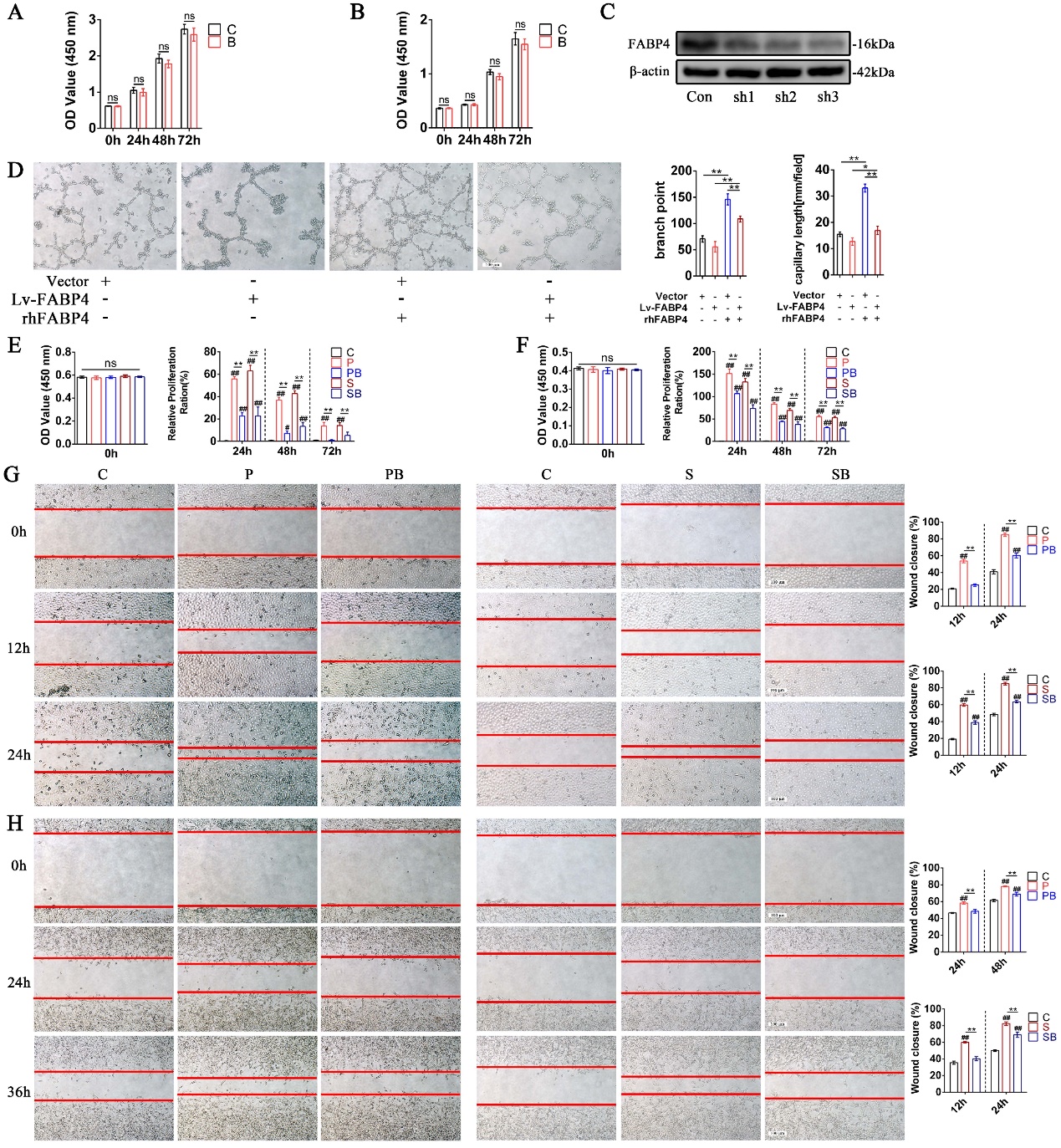


**Figure S3.** The role of FABP4 in HUVEC and FLS. **(A, B)** CCK-8 assay of HUVEC (A) and FLS (B) treated with C or B. The cell viability was measured based on the OD value (n = 3 per group). **(C)** Western blot of FABP4 in HUVEC treated with lentivirus mediated shRNA targeting FABP4. **(D)** Tube formation assay and quantification of HUVEC with lentivirus mediated shRNA targeting FABP4 (Lv-FABP4) transfection or rhFABP4 administration (n = 3 per group). Scale bar: 100 µm. **(E, F)** CCK-8 assay of HUVEC (E) and FLS (F) treated with C, P, PB, S, or SB. The cell viability and relative proliferation ratio were measured based on the OD value (n = 3 per group). **(G, H)** Wound-healing assays of HUVEC (G) and FLS (H) treated with C, P, PB, S, or SB. Scale bar: 100 μm. The statistical results of HUVEC and FLS wound-healing (n = 3 per group). One-way ANOVA and Tukey’s multiple comparison test. ^*^P < 0.05, ^**^P < 0.01; ^#^P < 0.05, ^##^P < 0.01 compared to the control; ns, not significant. Data are shown as the mean ± SEM.


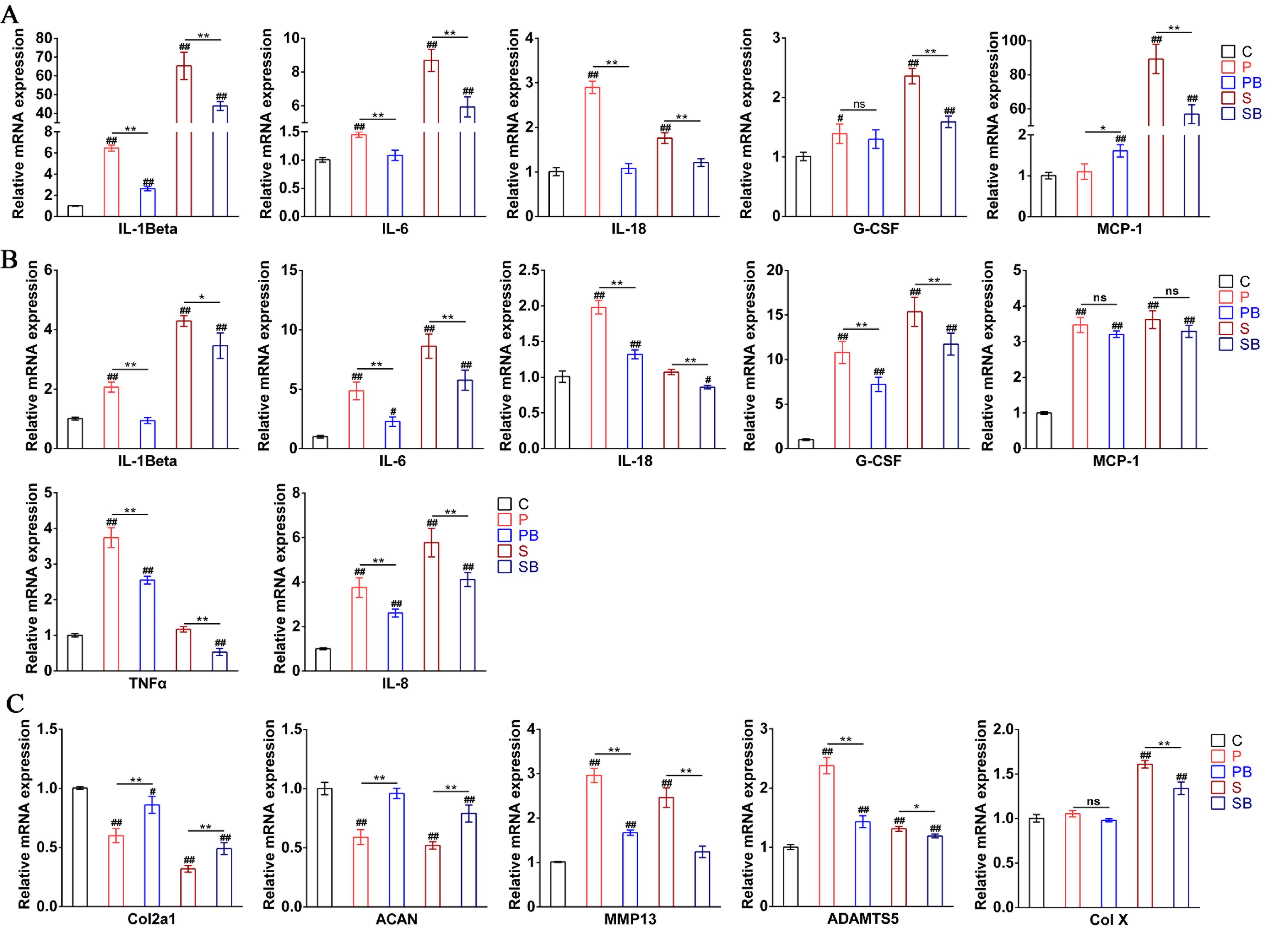


**Figure S4.** HUVEC, FLS and primary chondrocytes were stimulated with vehicle (C), rhFABP4 (P), rhFABP4 and BMS309403 (PB), M1-polarized macrophage supernatant (S), or M1-polarized macrophage supernatant and BMS309403 (SB) for 24 h. **(A)** Quantitative PCR analysis of IL-1β, IL-6, IL-18, G-CSF and MCP-1 mRNA expression in HUVEC (n = 3 per group). **(B)** Quantitative PCR analysis of IL-1β, IL-6, IL-18, G-CSF, MCP-1, TNFα and IL-8 mRNA expression in FLS (n = 3 per group). **(C)** Quantitative PCR analysis of Col2a1, ACAN, MMP13, ADAMT5 and Col X in primary chondrocytes (n = 3 per group). One-way ANOVA and Tukey’s multiple comparison test. ^*^P < 0.05, ^**^P < 0.01; ^#^P < 0.05, ^##^P < 0.01 compared to the control; ns, not significant. Data are shown as the mean ± SEM.


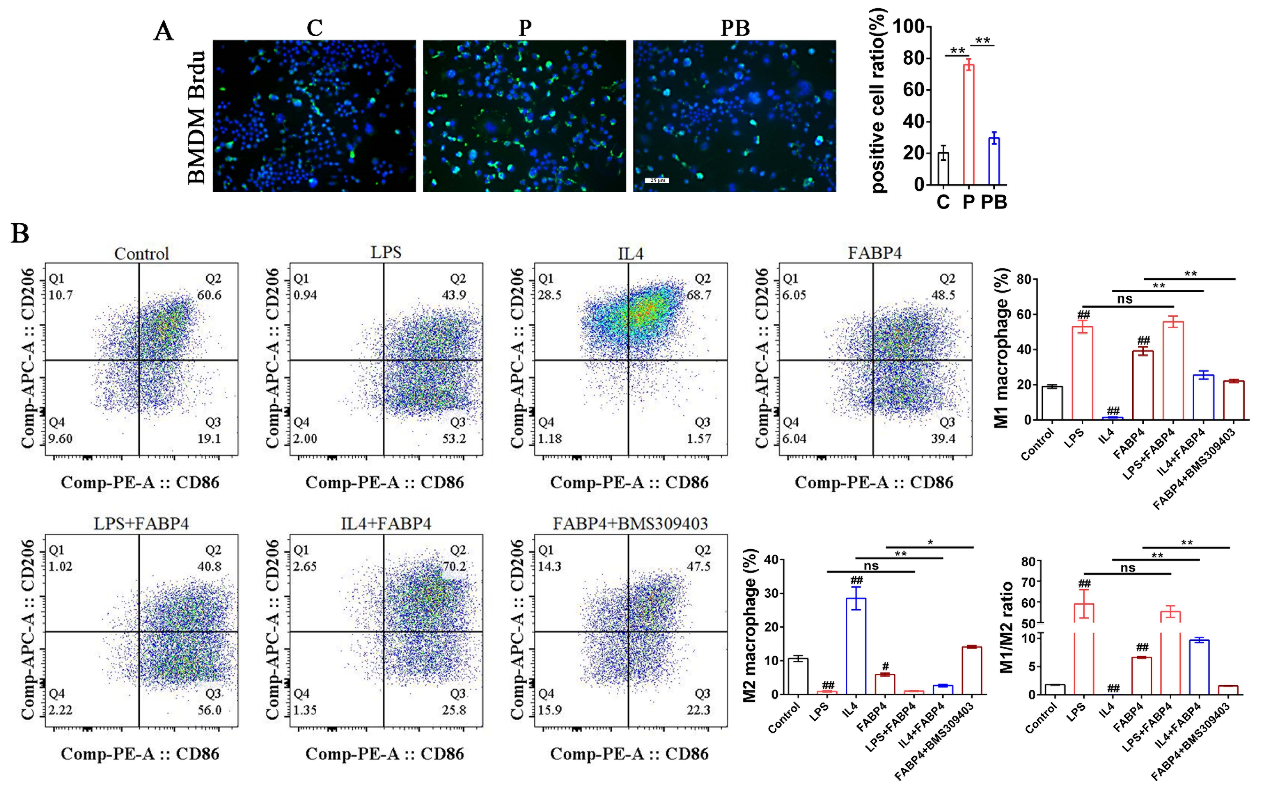


**Figure S5.** The effect of FABP4 on BMDMs *in vitro*. **(A)** Representative images and quantification of BrdU (green) immunofluorescence in BMDMs treated with C, P or PB for 24 hours (n = 3 per group). Scale bar: 25 µm. **(B)** Proportion of M1 and M2, and M1/M2 ratio in different treatment groups (n = 3 per group). One-way ANOVA and Tukey’s multiple comparison test. ^*^P < 0.05, ^**^P < 0.01; ^#^P < 0.05, ^##^P < 0.01 compared to the control; ns, not significant. Data are shown as the mean ± SEM.


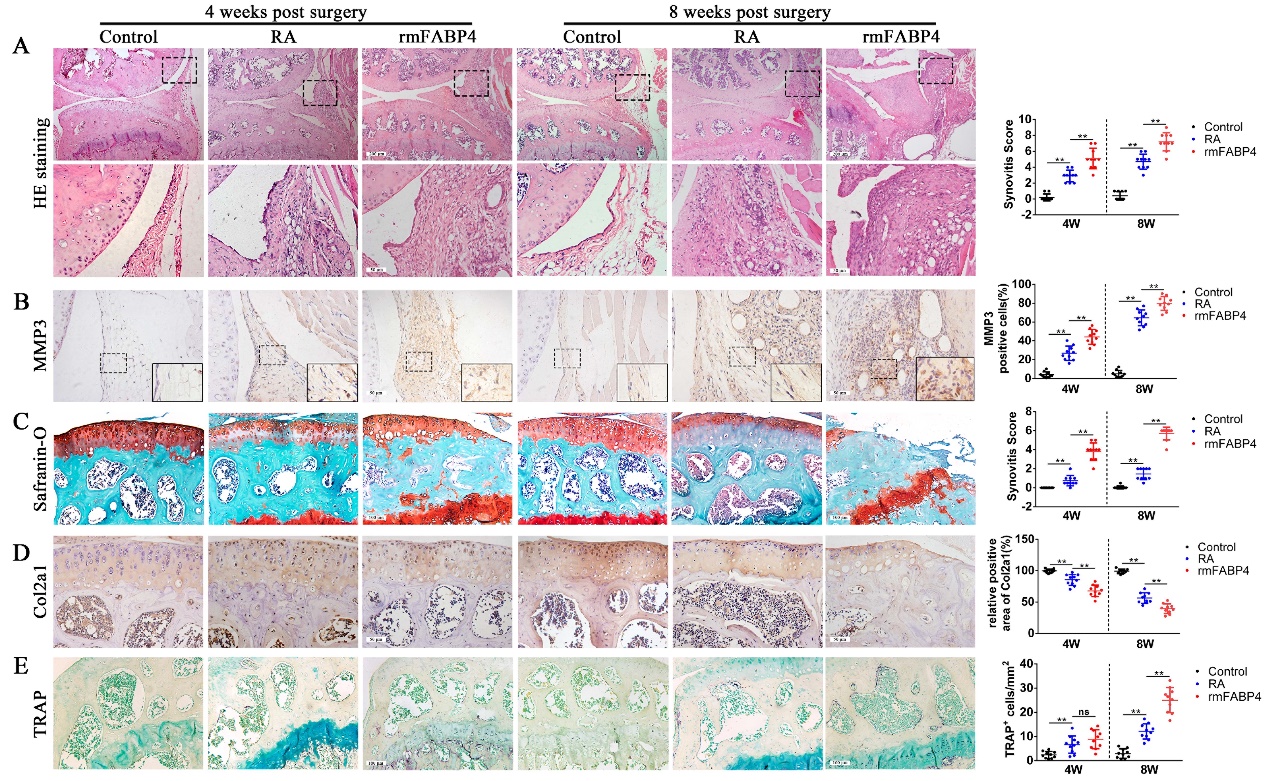


**Figure S6.** The phenotypes of C57BL/6 mice treated with rmFABP4 after AIA modeling. **(A-E)** Representative images and quantitative analysis of H&E staining (A), MMP3 immunohistochemical staining (B), Safranin O and Fast Green staining (C), Col2a1 immunohistochemical staining (D) and TRAP staining (E) in knee joints of controls and RA mice treated with a vehicle or rmFABP4 for 4 and 8 weeks (n = 10 per group). Scale bars: 50 µm, 100 µm and 200 µm. One-way ANOVA and Tukey’s multiple comparison test. ^**^P < 0.01; ns, no significance. Data are shown as the mean ± SEM.


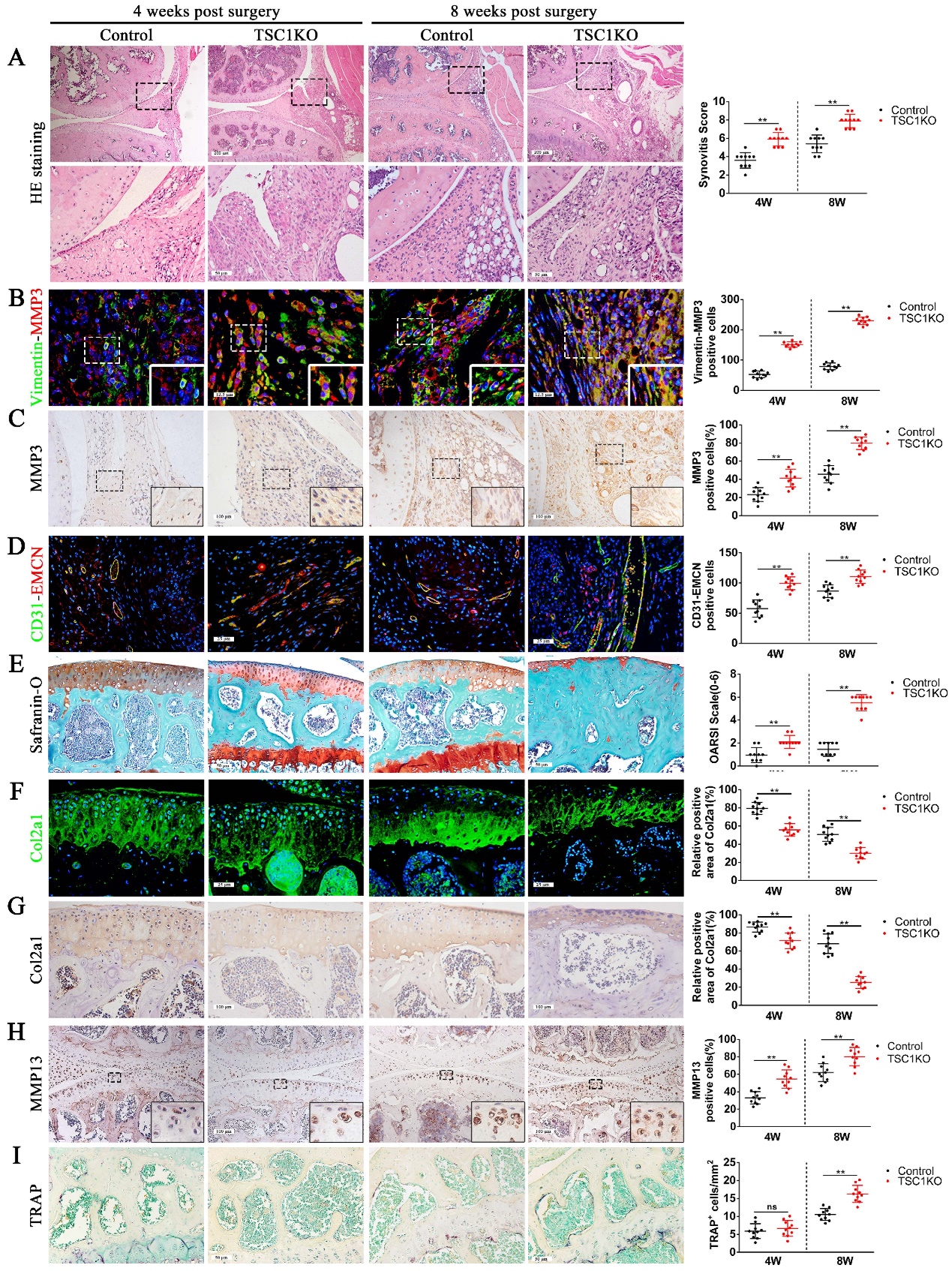


**Figure S7.** Phenotypes of TSC1KO mice after AIA modeling. **(A-I)** Representative images and quantification of H&E staining (A), Vimentin and MMP3 coimmunostaining (B), MMP3 immunohistochemical staining (C), CD31 and EMCN coimmunostaining (D), Safranin O and Fast Green staining (E), Col2a1 immunofluorescence staining (F), Col2a1 immunohistochemical staining (G), MMP13 immunohistochemical staining of (H) and TRAP staining (I) in the knee joints of controls and TSC1KO mice at 4 and 8 weeks (n = 10 per group). Scale bars: 12.5 µm, 25 µm, 50 µm, 100 µm and 200 µm. Student’s *t*-test. ^**^P < 0.01; ns, no significance. Data are shown as the mean ± SEM.


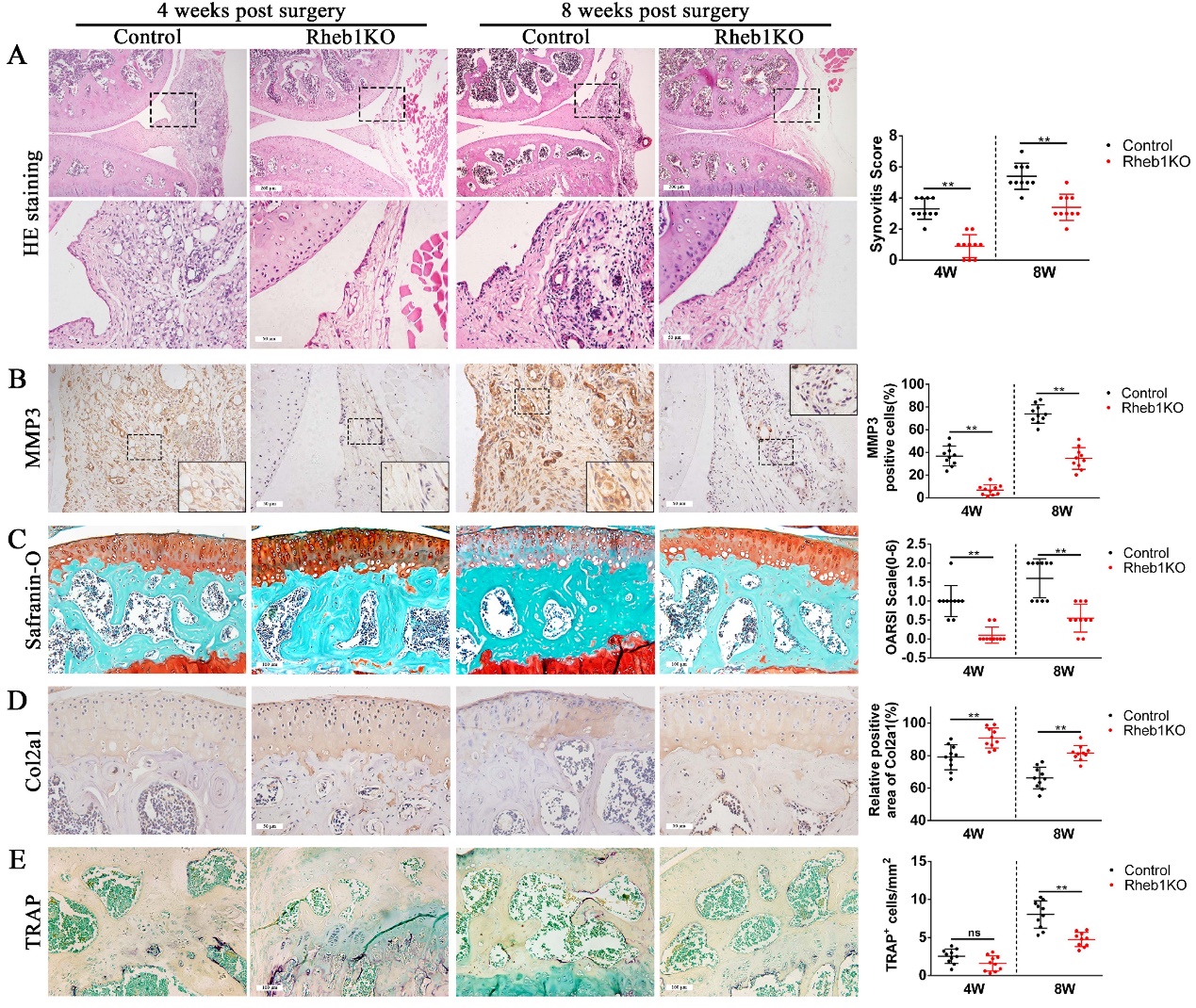


**Figure S8.** Rheb1KO mouse phenotypes after AIA modeling. **(A-E)** Representative images and quantitative analysis of H&E staining (A), MMP3 immunohistochemical staining (B), Safranin O and Fast Green staining (C), Col2a1 immunohistochemical staining (D) and TRAP staining (E) in the knee joints of controls and Rheb1KO mice at 4 and 8 weeks after AIA modeling (n = 10 per group). Scale bars: 50 µm, 100 µm and 200 µm. Student’s *t*-test. ^**^P < 0.01; ns, no significance. Data are shown as the mean ± SEM.


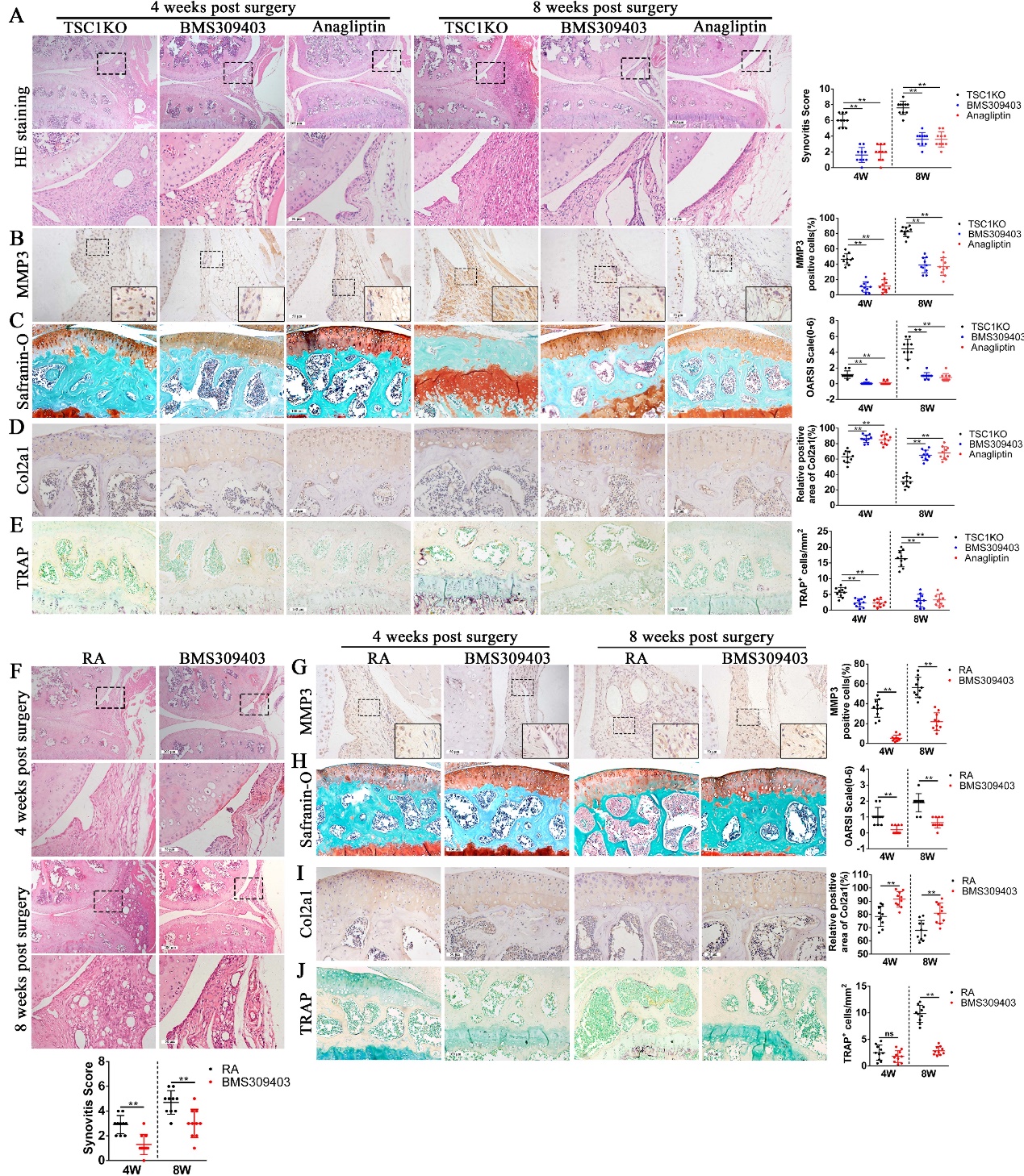


**Figure S9.** Phenotypes of RA mice treated with BMS309403 or anagliptin. **(A-E)** Representative images and quantitative analysis of H&E staining (A), MMP3 immunohistochemical staining (B), Safranin O and Fast Green staining (C), Col2a1 immunohistochemical staining (D) and TRAP staining (E) in the knee joints of TSC1KO mice treated with a vehicle, BMS309403 or anagliptin for 4 and 8 weeks after AIA modeling (n = 10 per group). Scale bar: 50 µm, 100 µm and 200 µm. **(F-J)** Representative images and quantitative analysis of H&E staining (F), MMP3 immunohistochemical staining (G), Safranin O and Fast Green staining (H), Col2a1 immunohistochemical staining (I) and TRAP staining (J) in the knee joints of C57BL/6 mice treated with a vehicle or BMS309403 for 4 and 8 weeks after AIA modeling (n = 10 per group). Scale bar: 50 µm, 100 µm and 200 µm. Student’s *t*-test or one-way ANOVA and Tukey’s multiple comparison test. ^**^P < 0.01; ns, no significance. Data are shown as the mean ± SEM.


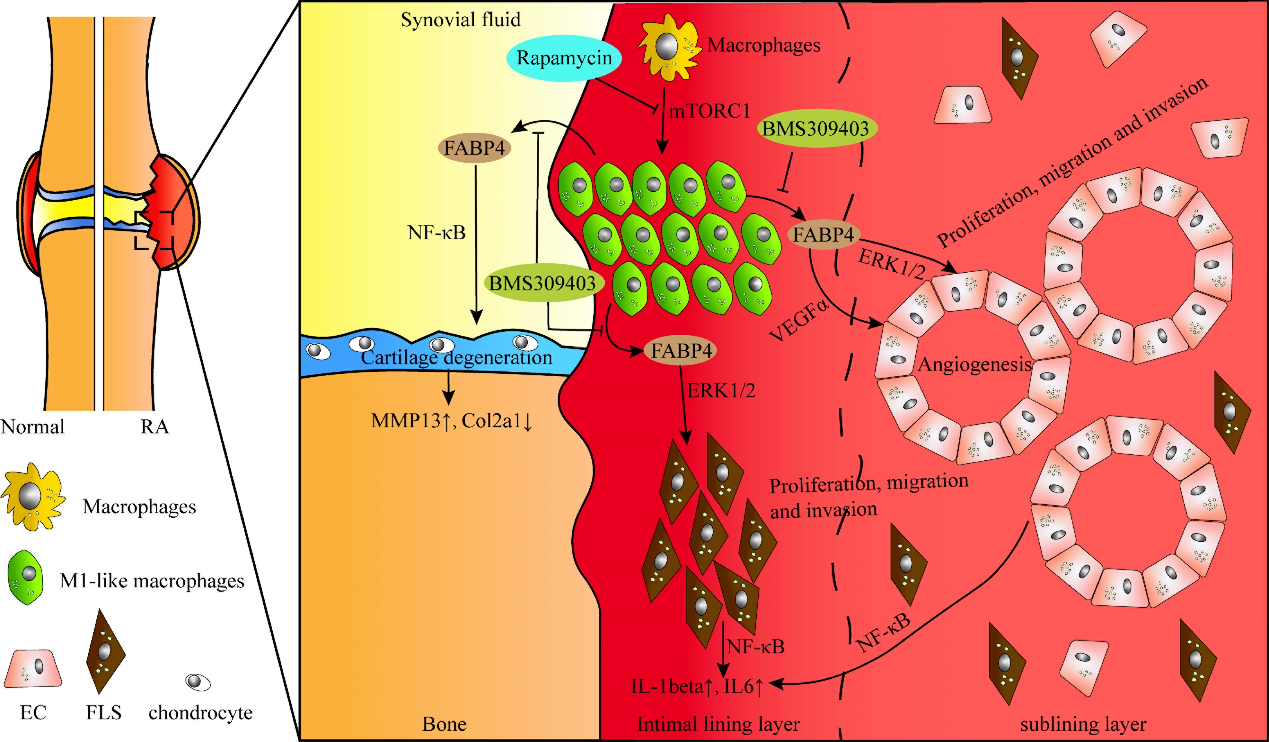


**Figure S10.** Model of FABP4 secretion by M1-polarized macrophages in promoting synovitis, angiogenesis and cartilage degeneration to aggravate RA progression.
